# An iron–oxygen intermediate formed during the catalytic cycle of cysteine dioxygenase†
†Electronic supplementary information (ESI) available: Experimental and computational details. See DOI: 10.1039/c6cc03904a



**DOI:** 10.1039/c6cc03904a

**Published:** 2016-06-09

**Authors:** E. P. Tchesnokov, A. S. Faponle, C. G. Davies, M. G. Quesne, R. Turner, M. Fellner, R. J. Souness, S. M. Wilbanks, S. P. de Visser, G. N. L. Jameson

**Affiliations:** a Department of Chemistry & MacDiarmid Institute for Advanced Materials and Nanotechnology University of Otago , PO Box 56 , Dunedin 9054 , New Zealand . Email: guy.jameson@otago.ac.nz; b Manchester Institute of Biotechnology and School of Chemical Engineering and Analytical Science , The University of Manchester , 131 Princess Street , Manchester M1 7DN , UK . Email: sam.devisser@manchester.ac.uk; c Centre for Free Radical Research , University of Otago , 2 Riccarton Ave , PO Box 4345 , Christchurch , New Zealand; d Department of Biochemistry , University of Otago , PO Box 56 , Dunedin 9054 , New Zealand

## Abstract

Cysteine dioxygenase is a key enzyme in the breakdown of cysteine, but its mechanism remains controversial. A combination of spectroscopic and computational studies provides the first evidence of a short-lived intermediate in the catalytic cycle. The intermediate decays within 20 ms and has absorption maxima at 500 and 640 nm.

## Abstract 2

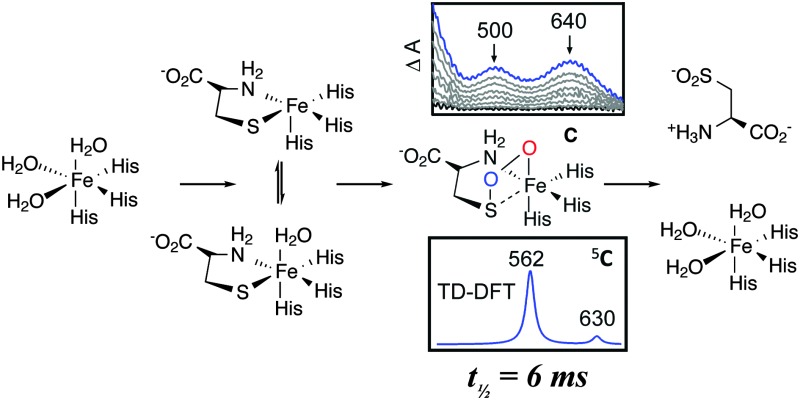
Combined spectroscopic, kinetic and computational studies provide first evidence of a short-lived intermediate in the catalytic cycle of cysteine dioxygenase.

Cysteine dioxygenase (CDO) catalyzes the first irreversible step in cysteine dissimilation through various pathways to taurine, pyruvate and sulfate.^1–3^ The substrate of CDO, cysteine, is a non-essential amino acid building block for protein biosynthesis and is an excitotoxic agent in the nervous system.^4,5^ The neuroexcitatory properties of cysteine implicate cysteine levels, and thus CDO function, in neurodegenerative diseases.^6,7^ However, recent findings by Brait *et al.* broadened this narrow appreciation of the pathological roles of cysteine and CDO by showing that forced expression of the CDO gene in human cancer cells markedly decreased tumor cell growth.^8^

CDO has unusual structural features that make it of great interest when trying to understand the factors that control iron–dioxygen chemistry.^9,10^ The octahedral ferrous iron is coordinated by three histidines rather than the more usual 2-His/1-carboxylate motif that is common in non-heme mononuclear iron enzymes. The neutral coordination of the protein observed in CDO is expected to affect individual reaction steps through stabilization of negative charge.^11^

The mechanism of CDO has been investigated computationally by one of us.^12,13^ These results showed that cysteine-bound CDO binds dioxygen to form an iron(iii)–superoxo species that attacks the bound sulfur of cysteine by the distal oxygen of superoxide (Scheme 1). Heterogeneous O–O bond cleavage leads to formation of an iron(iv)–oxo intermediate that supplies the second O atom to sulfur. This mechanism has been supported by combined spectroscopic and computational studies of small molecule biomimetic complexes of CDO some of which show formation of an iron(iv)–oxo intermediate.^14–16^ Here we report a single turnover approach allowing direct comparison of our experimental results to computational findings.

**Scheme 1:**
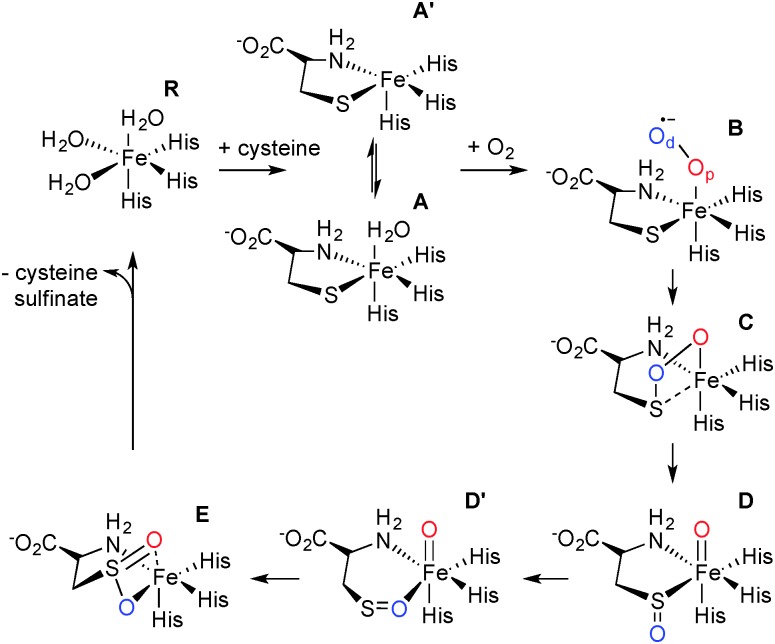
Catalytic cycle of CDO.

First the stability and spectroscopic description of the cysteine-bound complex was explored by Mössbauer spectroscopy. This was to find conditions under which single turnover experiments would be successful and to aid interpretation of freeze-quench experiments (*vide infra*) where the redox state of the iron during turnover could be probed. In this study we titrated cysteine with CDO at 20 and 4 °C and measured Mössbauer spectral changes. We were able to show that CDO forms a stoichiometric and stable active complex with cysteine.

Careful deconvolution of a large number of Mössbauer spectra (*n* = 17) showed that the signal corresponding to resting state CDO disappeared with concomitant formation of two new sub-spectra characterized by similar isomer shifts but differing quadrupole splittings (see ESI†). It can be seen that one of the species has parameters very similar to resting state CDO and was indeed wrongly attributed to unbound resting state CDO in our previous paper upon addition of 10 mM cysteine.^10^ We investigated whether the two sub-spectra could be caused by the presence or absence of the cysteine–tyrosine posttranslational crosslink found in close proximity to the iron.^9^ However, this is not the case. The C93G variant displays multiple turnover kinetics similar to wild-type enzyme^17^ and produces the same two Mössbauer sub-spectra as does wild-type CDO (ESI†) even though it is unable to form the crosslink. Indeed, observation of two Mössbauer sub-spectra upon substrate binding has been noted before for other non-heme mononuclear iron enzymes.^18,19^ Computational prediction of Mössbauer parameters has allowed us to assign the two sub spectra to cysteine bound CDO with (**A**) and without (**A′**) a bound water (Scheme 1 and ESI†). This designation is further supported by recent crystallographic evidence for (**A**) in a C164S variant of CDO.^20^

The proportion of cysteine-bound species was determined directly from the spectra and plotted *versus* the concentration of cysteine added, normalized to active site iron present (Fig. S4, ESI†). Fitting these data to standard equations yielded a CDO:Fe^II^:cysteine complex dissociation constant, *K*_D_, of 65 ± 20 μM. Because this is well below the concentration of CDO and cysteine used in data collection, it represents an upper limit but confirms relatively tight binding of cysteine. These samples were then exposed to air to check that they were catalytically competent by monitoring the appearance of cysteine sulfinic acid (CSA) product by HPLC and MS analysis (ESI†). The amount of CSA produced was stoichiometric with respect to the concentration of CDO:Fe^II^:cysteine complex.

The production and isolation of a catalytically active cysteine-bound species allowed the dioxygenation reaction of cysteine dioxygenase to be studied. We initially measured UV/vis spectral changes of anaerobic CDO:Fe^II^:cysteine complex after mixing with degassed and oxygenated buffer but only observed changes when the temperature was lowered to 4 °C (Fig. 1a). No spectral changes could be observed after mixing with degassed buffer showing that our procedures were strictly anaerobic. However, CDO:Fe^II^:cysteine complex mixed with oxygenated buffer exhibited formation of two absorption maxima (500 and 640 nm) and a shoulder at 350 nm within the mixing time of the apparatus (∼1 ms). The absorption was proportional to the amount of enzyme used and dioxygen added. These absorption features disappeared within approximately 20 ms with a first order rate constant of 112 ± 5 s^–1^ which is independent of oxygen concentration (Fig. 1b–e). This indicated that the species is an oxygen adduct of CDO.

**Fig. 1:**
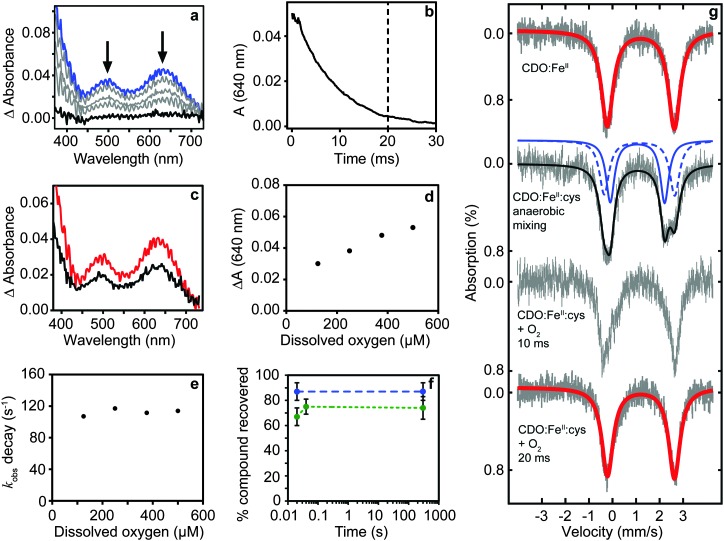
Production of a catalytically competent intermediate during single turnover of CDO. (a) Transient absorption spectrum of the intermediate formed within mixing (blue) disappears within 20 ms (b). The amount of intermediate is dependent upon the amount of protein (500 (red) and 240 μM (black) CDO:cysteine complex) (c) and dioxygen (d). The rate of decay is independent of the dioxygen concentration (e). Chemical quench with HCl shows ∼85% cysteine (blue) is recovered under anaerobic mixing while most CSA (∼70%, green) is produced within 20 ms suggesting the intermediate is on-pathway (f). Mössbauer spectrum of the Fe(ii) resting state; anaerobic mixing followed by freeze-quench recovers the cysteine bound ES complex; freeze-quench after mixing with dissolved oxygen recovered the Fe(ii) resting state (g).

Chemical quench experiments using HCl as quenching agent allowed us to investigate whether the intermediate was on-pathway (Fig. 1f). Two independent experiments involving different enzyme preparations showed no CSA could be detected in CDO:Fe^II^:cysteine containing samples quenched anaerobically, while the recovery of unused cysteine substrate was near stoichiometric (87 ± 7%, *n* = 7). CDO:Fe^II^:cysteine mixed with oxygenated buffer for 20 ms and quenched showed high conversion to CSA (67 ± 7%, *n* = 5). Importantly, unreacted cysteine was not detectable strongly supporting complete turnover. Indeed, the slightly lower value of conversion is probably caused by precipitation of CSA during acid quenching and this is supported by essentially identical recoveries from samples quenched after longer (minute) reaction times (74 ± 9%, *n* = 3).

The reaction was further investigated by freeze-quench studies. Mixture of anaerobic cysteine-bound CDO with stoichiometric amounts of dioxygen (supplied as oxygenated buffer) at 4 °C followed by quenching through freezing in 2-methylbutane after 10 and 20 ms gave Mössbauer spectra consistent with pure resting state CDO (Fig. 1g). The return of the chemical system to resting state within a time period of 20 ms implicates a fast dioxygenation step with a second order reaction rate constant of ≥3 × 10^5^ M^–1^ s^–1^ and quite possibly even faster.

To confirm that turnover occurred in these samples, they were subsequently thawed under strict anaerobic conditions inside the glove box. Thereafter, the sample was tested for the presence of CSA and near stoichiometric amounts of CSA were determined by mass spectrometry (652 μM CDO:Fe^II^:cysteine produced 573 μM CSA, 88% conversion). In a completely independent replicate experiment involving an entirely different enzyme preparation, a 92% turnover was determined using HPLC-evaporative light scattering (ELSD) analysis.

Unfortunately, our attempts to definitively spectroscopically characterize the colored intermediate by other means were unsuccessful. Freeze-quench down to ∼10 ms did not show Mössbauer spectra widely different from resting state and our C93G variant showed a similar intermediate that decayed even faster (see ESI†). Importantly, however, our studies highlight that presence of cysteine 93 as a free thiol (rather than being crosslinked with tyrosine 157) slows the reaction. This is consistent with our previous investigations^17^ and explains why crosslink formation increases activity.

In order to gain insight into the structure and properties of this short-lived intermediate, we decided to use DFT and *ab initio* (CASSCF/NEVPT2) methods to probe possible candidates. Our computational methods were calibrated and benchmarked previously and shown accurately to reproduce experimental resonance Raman spectra, infrared spectra as well as kinetic isotope effects and rate constants excellently.^21,22^ We initially took the DFT optimized geometries of catalytic cycle intermediates with two oxygen atoms included from previous studies^12^ and after reoptimizing with the methods described in the ESI† analyzed the absorption spectra for each species in the triplet and quintet spin states using various methods and basis sets. The focus of the calculations was on the iron(iii)–superoxo (**B**), iron(iii)-bicyclic ring-structure (**C**), iron(iv)–oxo–cysteinesulfoxide complex (**D** and **D′**) as well as the iron(iii)–cysteine–sulfinic acid product complex (**E**). To complement the series and confirm that the signals correspond to post-oxygen binding, also several catalytic cycle intermediates not previously considered yet were investigated, *e.g.* the **A** and **A′** described above. In addition, CASSCF(10,14)/NEVPT2/BS2 calculations on ^5^ **B** and ^5^ **C** were performed.

Fig. 2 shows calculated absorption spectra of key catalytic intermediates and the potential energy landscape. The rate determining barrier is the attack of the superoxo on sulfur to form a bicyclic ring structure with an activation barrier of 10.0 kcal mol^–1^ (Δ*G* ^‡^ = 13.4 kcal mol^–1^) in the quintet spin state. Note that the first two reaction barriers are relatively high in energy (10.0 and 8.2 kcal mol^–1^ with respect to their precursors) and as such both ^5^ **B** and ^5^ **C** should have a finite lifetime, whereas all other quintet intermediates will be short-lived and most likely too short to detect. As such, based on the energy landscape there are only two likely candidates for the UV/Vis absorption features in Fig. 1, namely ^5^ **B** and ^5^ **C** as they are located in a potential well.

**Fig. 2:**
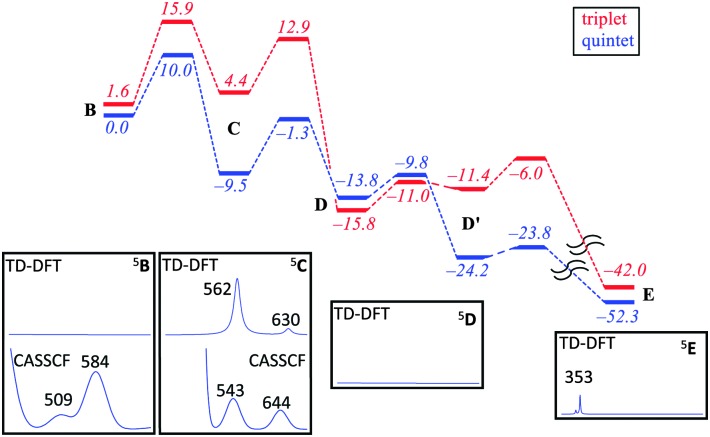
Potential energy profile with energies in kcal mol^–1^ of cysteine dioxygenation by CDO models as calculated with B3LYP/BS2//B3LYP/BS1. Insets show calculated (TD-DFT/BS2 and CASSCF/BS2) absorption spectra of local minima.

An analysis of the TD-DFT calculated absorption spectra of all species shows that very few structures give patterns in the 400–700 nm range that match the experimental spectrum of Fig. 1a. However, there is some degree of variation between different methods and basis sets. Nevertheless with all methods applied we find several absorption bands in the 400–700 nm range for the bicyclic ring-structure (**C**) in the quintet spin state. This structure gives maxima in the absorption spectrum at 552 and 661 nm with TD-DFT/BS1, 562 and 630 nm with TD-DFT/BS2 and 543 and 644 nm with CASSCF(10,14)/BS2; all of which are in good qualitative agreement with the maxima seen in Fig. 1a and are consistent with the fact that DFT tends to overestimate absorption and frequency bands by 5–10%. These bands refer to excitation of an electron from the π*_OO,*x*_ orbital into the S–O antibonding orbital (562 nm transition) or from the π*_yz_ orbital on iron to the O–O antibonding (σ) orbital (630 nm transition). In contrast, no absorption maxima between 400 and 700 nm could be found for the iron(iii)–superoxo structure, the cysteine sulfoxide (**D** and **D′**) nor the cysteine sulfinic acid (**E**).

Despite the fact that the TD-DFT calculated spectra of the ferric superoxide ^5^ **B** give no significant absorption patterns in the 400–700 nm range, at CASSCF level of theory two bands appear, although significantly shifted with respect to those obtained experimentally. Therefore, although we cannot rule ^5^ **B** out entirely, the most likely structure that contributes to the experimental absorption spectrum is the bicyclic ring structure in the quintet spin state (**C**). We tried to confirm this by other means. Freeze-quench down to 10 ms shows a Mössbauer spectrum that is very close to resting state (Fig. 1g). Calculated Mössbauer parameters (ESI†) for all the species suggests that both the ferric superoxide and bicyclic system have similar Mössbauer parameters to resting state and thus, unfortunately, the current data is not conclusive but describes our current best understanding.

The typical 3-His coordination of CDO, rather than the 2-His/1-carboxylate ligand system in most non-heme iron dioxygenases may be responsible for the enhanced stability of the bicyclic ring structure and its relative large barrier to convert to an iron(iv)–oxo intermediate. This may give it a slightly longer lifetime in CDO than in alternative non-heme iron dioxygenases. For example, a related bicyclic intermediate in homoprotocatechuate 2,3-dioxygenase (2,3-HPCD) has been observed crystallographically but not spectroscopically characterized.^23,24^ Likewise, in TauD^25,26^ the barrier to O–O bond scission is 3.6 kcal mol^–1^ and this explains why this intermediate is not observed during freeze-quench studies.^27^

The fact that an intermediate is observable in CDO, although only transiently, suggests that through suitable modification of the active site this intermediate might be further stabilized and such studies are underway in our laboratories. With observation of this intermediate we have experimental evidence to support the mechanism of CDO previously proposed.^12,13^ We also have initial spectroscopic evidence for iron–oxygen attack on a thiol at a non-heme mononuclear-iron enzyme.

GNLJ thanks the Marsden Fund of the Royal Society of New Zealand for funding. EPT thanks the Canadian Institutes of Health Research for a postdoctoral fellowship. RJS was the recipient of a TEC Top Achiever Doctoral Scholarship. SdV thanks the National Service of Computational Chemistry Software (NSCCS) for cpu time. ASF and MGQ thank the Tertiary Education Trust Fund and the BBSRC for studentships. B. Zhang, C. Pollock, J. M. Bollinger Jr. and C. Krebs of Penn State are thanked for freeze-quench studies down to 10 ms.
