# Supplementary material for: An iron–oxygen intermediate formed during the catalytic cycle of cysteine dioxygenase
Source: Chem Commun (Camb). 2016 Jun 9;52(57):8814–7. doi: 10.1039/c6cc03904a (PMC5043143; doi:10.1039/c6cc03904a)
Supplement: Supplementary file 1 [file CC-052-C6CC03904A-s001.pdf]

Electronic Supplementary Material (ESI) for ChemComm.
This journal is © The Royal Society of Chemistry 2016

Electronic Supplementary Information

An Iron-Oxygen Intermediate Formed During the Catalytic Cycle of Cysteine Dioxygenase

E. P. Tchesnokov,[a] A. S. Faponle,[b] C. G. Davies,[a] M. G. Quesne,[b] R. Turner,[c] M. Fellner,[a] R. J. Souness,[a] S. M. Wilbanks,[d] S. P. de Visser*,[b] and G. N. L. Jameson*,[a]

[a] Department of Chemistry & MacDiarmid Institute for Advanced Materials and Nanotechnology University of Otago, PO Box 56, Dunedin 9054, New Zealand.

[b] Manchester Institute of Biotechnology and School of Chemical Engineering and Analytical Science, The University of Manchester, 131 Princess Street, Manchester M1 7DN, United Kingdom.

[c] Centre for Free Radical Research, University of Otago, 2 Riccarton Ave, PO Box 4345, Christchurch, New Zealand

[d] Department of Biochemistry, University of Otago, PO Box 56, Dunedin 9054, New Zealand.

Contents

1. **Protein purification**
2. **Mössbauer spectroscopy**
3. **Stopped-flow**
4. **Freeze-quench**
5. **Chemical-quench**
6. **CSA determination**
7. **Formation of an active CDO:cysteine substrate complex**
8. **Kinetic investigation of the short-lived intermediate**
9. **Freeze-quench study of single turnover**
10. **Computational methods**

1. Protein purification

The rat CDO coding sequence was kindly provided by Dr. Martha H. Stipanuk (Cornell University)[1]. CDO was expressed and purified using Strep-tag affinity technology (IBA)[2] as previously reported by our group[3]. The purified protein preparations were dialyzed extensively (>10^9 dilution factor) against buffer containing 100 mM Tris-HCl (pH 8.0) and 50 mM NaCl. Protein was concentrated on Vivaspin centrifugation concentrators (GE Healthscience) to the appropriate concentrations for the subsequent experiments. Protein concentrations were determined spectrophotometrically at 280 nm using an experimentally derived extinction coefficient of 38,100 $M^{-1}cm^{-1}$.[3] Consistent with previous observations[4], CDO preparations resolved into two bands when subjected to SDS-PAGE revealing the fraction of the protein with C93-Y157 crosslink as a result of a posttranslational modification (Figure S1).

Protein preparations, corresponding buffers and solvents were made anaerobic by alternating cycles of vacuum and purging with argon on a Schlenk line with the Schlenk tube kept on an ice slurry. All anaerobic manipulations were performed in a nitrogen atmosphere in a glovebox (Belle Technology).

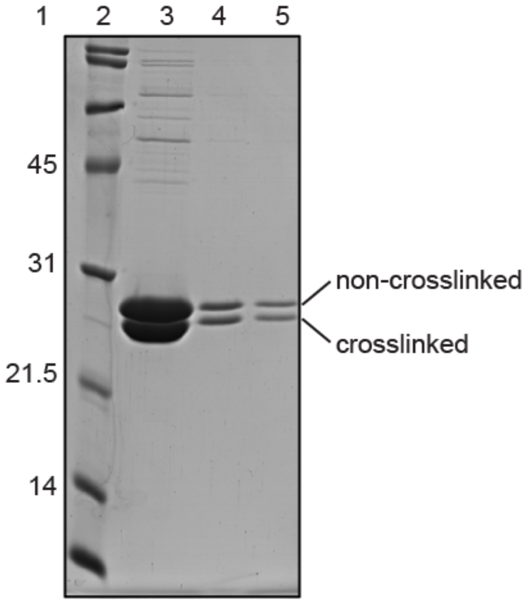

Figure S1. SDS-PAGE analysis of purified rat CDO. Purified samples of rat CDO were resolved by SDS-PAGE on a 15% (w/v) polyacrylamide gel and detected by staining with Coomassie Blue dye. *Lane 1*, molecular weight (kDa) of selected markers. *Lane 2*, migration pattern of molecular weight markers. *Lane 3*, purified wild type rat CDO (7.7 µg). *Lane 4*, same as *Lane 3*, except 20 times less of protein amount was loaded. *Lane 5*, same as *Lane 3*, except 40 times less of protein amount was loaded.

2. Mössbauer spectroscopy

Ferrous iron stock solutions for Mössbauer spectroscopy experiments were prepared as previously described.[5] Anaerobic preparations of protein (1-1.4 mM) in 100 mM Tris-HCl (pH 8.0) and 50 mM NaCl were iron-saturated anaerobically by exogenously adding ferrous iron in the presence of 1 equiv of dithionite followed by treatment with Analytical Grade Chelex 100 sodium form, 200-400 mesh (Bio-Rad). Protein samples were frozen in liquid nitrogen in a glovebox (Belle Technology). ^{57}Fe Mössbauer spectra of frozen liquid samples in a custom teflon sample holder (approximately 400 µL volume) were recorded on a Mössbauer spectrometer from

SEE Co. (Science Engineering & Education Co., MN) equipped with a closed-cycle refrigerator system from Janis Research Co. and SHI (Sumitomo Heavy Industries Ltd.) and a temperature controller from Lakeshore Cryotronics, Inc. Data were collected in constant acceleration mode in transmission geometry with an applied field of 47 mT parallel to the γ-rays. Mössbauer spectrum of the 10-ms rapid freeze-quenched was initially recorded at 4.2 K on an alternating constant acceleration Mössbauer spectrometer equipped with a Janis SVT-400 variable-temperature cryostat at Penn State. In this case, the spectrum was recorded in the absence of an externally applied magnetic field. The zero velocity of the Mössbauer spectra refers to the centroid of the room temperature spectrum of a 25 μm metallic iron foil.

3. Stopped-flow

SX-20MV stopped-flow spectrometer with Xe-arc lamp (Applied Photophysics) was used to mix anaerobic CDO:cysteine complex (~1.0 equiv of cysteine) with oxygenated buffer (1:1) at 4 °C. Temperature was controlled by a refrigerated, closed-circuit water bath (DC10-K10, Haake). Prior to the experiment the apparatus drive syringes and flow circuits were washed with 3 mM dithionite solution and rinsed extensively with deoxygenated buffer. Kinetic experiments were completed in single mixing mode and followed using a photo-diode array or photomultiplier tube. Single wavelength kinetic traces (500 and 640 nm) were fitted with the SX20 Pro-Data Viewer software (Applied Photophysics). Spectral changes in the region 380-740 nm were analyzed using PC Pro-K Global analysis and Data simulation software (Applied Photophysics). Further analysis and presentation were completed using Excel (Microsoft) and Prism 5 (GraphPad).

4. Freeze-quench

SFM-300 (BioLogic, Science Instruments) was used to asymmetrically mix CDO:cysteine complex with oxygen saturated buffer (2:1) at 4°C or 25°C. Temperature was controlled by a refrigerated, closed-circuit water bath (RW-0525G, Jeio Tech). Reported incubation times were generated using SFM-300 (BioLogic, Science Instruments) settings and the accompanying Bio-kine 4.45 software (BioLogic, Science Instruments). The samples were frozen in 2-methylbutane (Sigma-Aldrich) kept at -120°C using a liquid nitrogen bath. The reported reaction time was calibrated by following complex formation of ferrous iron (1 mM) with ferrozine (6 mM) at λ = 562 nm and by freeze quench Mössbauer under the same conditions. At 4°C complex formation has a rate of 19.2 ± 0.2 s^{-1} using a 0.08 cm path-length cell to allow observation of the highly colored complex (ε = 27,900 $M^{-1}cm^{-1}$). In the freeze-quenched samples, the low spin complex is easily identified and the proportion determined from the Mössbauer spectrum measured at 4.5 K. Comparison of the data from the two methods shows that all freeze-quench samples under these conditions are in fact 10 ms older than their mixing time, indicating the time of freezing (see below). To ensure correct mixing with the 2:1 mixing ratio, the fastest mixing time possible is 10 ms and thus our fastest quench time is 20 ms.

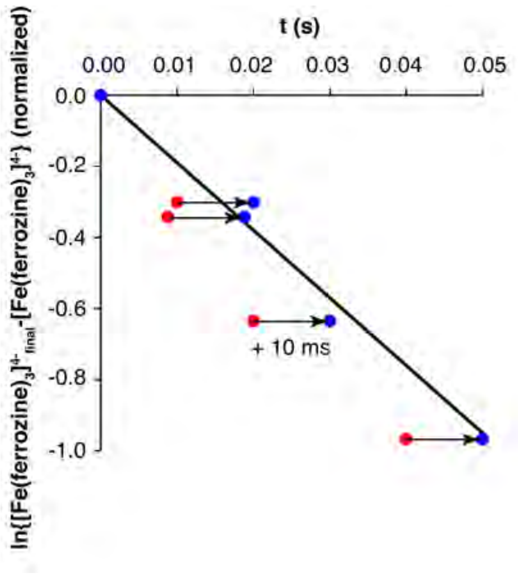

Figure S2. Plot of ln(complex$_{final}$ – complex) versus time for the reaction of ferrous iron with ferrozine. The black line represents a rate of 19.2 s^{-1} observed by following λ=562 nm. The red circles are obtained from freeze-quench Mössbauer samples using the mixing time to determine the age of the sample. Addition of 10 ms to each time point leads to the blue points that overlay the absorption data. Thus the freezing time is determined to be 10 ms.

For the 10-ms freeze-quench, an anaerobic solution containing CDO:cysteine complex (800 uM ^{57}Fe and 1.0 eq. of cysteine) was mixed with an oxygen-saturated buffer (~ 1.8 mM O_2) in a 2:1 (v:v) ratio for 10 ms before frozen in cold 2-methylbutane bath (-160 °C). The general procedure and the particular freeze-quench apparatus have been published previously.[6]

5. Chemical-quench

SFM-300 (BioLogic, Science Instruments) was used to mix anaerobic CDO:cysteine complex (~1.0 eq of cysteine) with degassed and oxygenated buffer (1:1) at 4°C for 20 and 40 ms followed by mixing with extensively degassed HCl (1:1). The apparatus was made anaerobic the same way as described for freeze-quench above. Reported incubation times were generated using SFM-300 (BioLogic, Science Instruments) settings and the accompanying Bio-kine 4.45 software (BioLogic, Science Instruments). Samples were subjected to HPLC-ELSD analysis in order to determine cysteine and CSA concentrations. CDO:cysteine complex was prepared in 100 mM phosphate buffer (pH 7.5) to avoid HILIC column (Phenomenex) poisoning. Concentration of cysteine and CSA in end point reaction (>5 minutes) samples was used to determine percent recovery of cysteine substrate in samples mixed with degassed buffer and percent turnover of CSA product in samples mixed with oxygenated buffer. Protein was precipitated with a 9-fold excess of acetone or acetonitrile to the sample solution. Samples were then spun and the supernatant collected and analyzed by either HPLC or LCMS for quantification of cysteine sulfinic acid (see below). CSA concentrations were found to be slightly lower than under normal assay conditions even when dioxygen was unlimited and we believe this is due to some precipitation during quenching by HCl.

Table S1. Chemical-quench data.

Mixing time	[CDO:cysteine] Final after mixing (µM)	Mixing	[cysteine] Measured (µM)	% cysteine recovered	± Standard error	*n*	[CSA] Measured (µM)	% CSA produced	± Standard error	*n*
20 ms	690	anaerobic	550	80		2	not detectable			
20 ms	680	anaerobic	645	95		4	not detectable			
> 5 min	690	anaerobic	590	86		1	not detectable			
			**Overall**	**87**	**7**	**7**				
20 ms	345	aerobic	not detectable				237	69		3
20 ma	340	aerobic	not detectable				220	65		2
40 ms	345	aerobic	not detectable				285	83		2
40 ms	340	aerobic	not detectable				230	68		1
> 5 min	345	aerobic	not detectable				260	75		1
> 5 min	340	aerobic	not detectable				250	74		2
							**Overall 20 ms**	**67**	**7**	**5**
							**Overall 40 ms**	**75**	**6**	**3**
							**Overall > 5 min**	**74**	**9**	**3**

6. CSA determination

CSA was determined either by HPLC[7] as described previously or LCMS. LC-ESI-MS/MS analyses were performed in the negative ion mode with an Applied Biosystems API 4000 QTRAP mass spectrometer. A Luna HILIC column (150 mm × 2.0 mm, 3 µm) was used. 20 µl of sample was injected. Solvent A was acetonitrile and Solvent B was ammonium acetate 20 mM, pH=7.0. Starting conditions were 10% B for 3 minutes then a gradient to 50% B over 5 minutes returning to starting conditions and re-equilibration. The electrospray needle was held at -5000 V at 400°C. Dinitrogen was used as a sheath gas and set at 10 units. The collision gas was dinitrogen with a collisional energy of 53 arbitrary units. Multiple reaction monitoring was performed and two product ions with *m/z* of 64 and 88 from the precursor ion 151.9 were measured. The *m/z* 64 ion was used for quantification and the *m/z* 88 ion was used for confirmation. A standard curve was prepared from 0.1 to 500 µM of cysteine sulfinic acid.

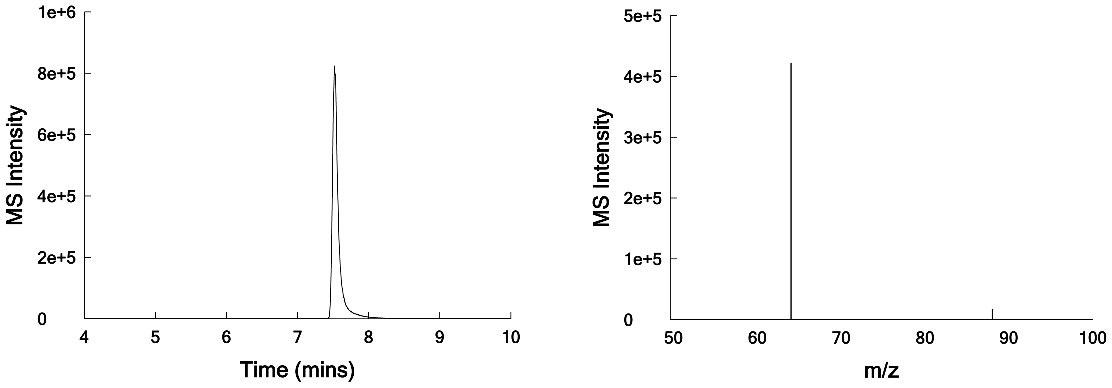

CSA Chromatogram monitoring 151.9 to 64 | MS2 Fragmentation pattern of 151.9

Figure S3. Typical chromatogram and MSMS fragmentation during CSA quantification of CDO enzyme assays.

7. Formation of an active CDO:cysteine substrate complex

Anaerobic CDO (~1 mM active site ferrous iron in 100 mM Tris-HCl (pH 8.0) and 50 mM NaCl) was mixed with cysteine substrate either manually in the glovebox (Belle Technology) at 20 °C or mechanically in a SFM-300 (BioLogic, Science Instruments) at 4 °C. The range of cysteine concentrations extended below and above CDO active site ferrous iron concentration. Samples prepared in the glovebox were frozen anaerobically in liquid nitrogen within 90 seconds of cysteine addition. Samples prepared using SFM-300 (BioLogic, Science Instruments) were frozen in 2-methylbutane (as described further in the Rapid Freeze Quench Methods (RFQ) section). All RFQ samples (including the resting state) used to monitor the formation of CDO:cysteine substrate complex were generated within the same sequence of shots.

Determination of CDO:cysteine Complex Dissociation Constant (K_d).[8] Formation of the *ES* complex as a function of substrate concentration was followed using Mössbauer spectroscopy and substrate bound fraction was determined assuming that the Lamb-Mössbauer factors of bound and non-bound protein were the same. The fraction bound was plotted against cysteine concentration normalized to active site ferrous iron concentration present in the sample. Consequently, a modified version of the equation describing ES complex formation under conditions of substrate depletion (tight binding) was derived:

$$\frac{[ES]}{[E]} = \frac{1}{2}(K+1+x) - \sqrt{\frac{1}{4}(K+1+x)^2 - x} \qquad (1)$$

$$K = \frac{K_d}{[E]}; \quad x = \frac{[S]}{[E]}$$

For data analysis ordinate values were calculated as $y = [ES]/[E]$ and abscissa values were calculated as $x = [S]/[E]$. Data from eight independent experiments with similar enzyme concentrations (weighted average = 884 μM) were used to determine the ES dissociation constant (K_d).

Cysteine binds stoichiometrically to form a catalytically competent ES complex. To investigate the binding properties of cysteine, CDO was titrated with cysteine at 20°C and 4°C and Mössbauer spectra measured (Figure S3). Addition of cysteine led to the gradual conversion of the symmetrical quadrupole doublet indicative of resting state high spin Fe^{II} into a more complicated, broader signal, similar to that observed previously[3]. Spectral changes ceased to occur at greater than stoichiometric concentrations of substrate and we concluded that at suprastoichiometric cysteine concentrations the CDO active site is saturated with substrate, indicating that cysteine binding is stoichiometric. Careful comparison of the CDO ES spectra showed that the signal corresponding to resting state CDO disappeared (solid red line) with the concomitant formation of two new species (solid and dashed blue lines) characterized by similar isomer shifts but differing quadrupole splittings. However, all species have parameters indicative of high spin iron(II) and therefore do not reflect a change in oxidation state. To confirm the presence of two cysteine-bound species, a large number of spectra (n = 17) were fitted independently and average parameters consistent with all spectra determined. It can be seen that one of the species has parameters very similar to resting state CDO (Table S2).

Deconvolution of the Mössbauer spectra of CDO titrated with cysteine substrate was achieved by fitting all available data to a single parameter file where isomer shift, quadrupole splitting and line width were constrained to the aforementioned average values (Table S2), while only relative areas were allowed to vary. Since the two forms were both present in similar proportions (60:40) under all cysteine concentrations they were treated as a single species for the purposes of calculating K_d. The proportion of cysteine-bound species was determined directly from the spectra and plotted versus the concentration of cysteine added, normalized to active site iron present. Under substoichiometric cysteine concentrations, the ES complex formation was stoichiometric to total cysteine added. This suggested that under these conditions cysteine binding is strongly in favor of ES complex formation at either 20°C or 4°C (Figure S4). Fitting these data to standard equations yielded a CDO-cysteine complex dissociation constant, K_d, of 65 ± 20 μM. As this is well below the concentration of CDO and cysteine used in data collection, it represents an upper limit.

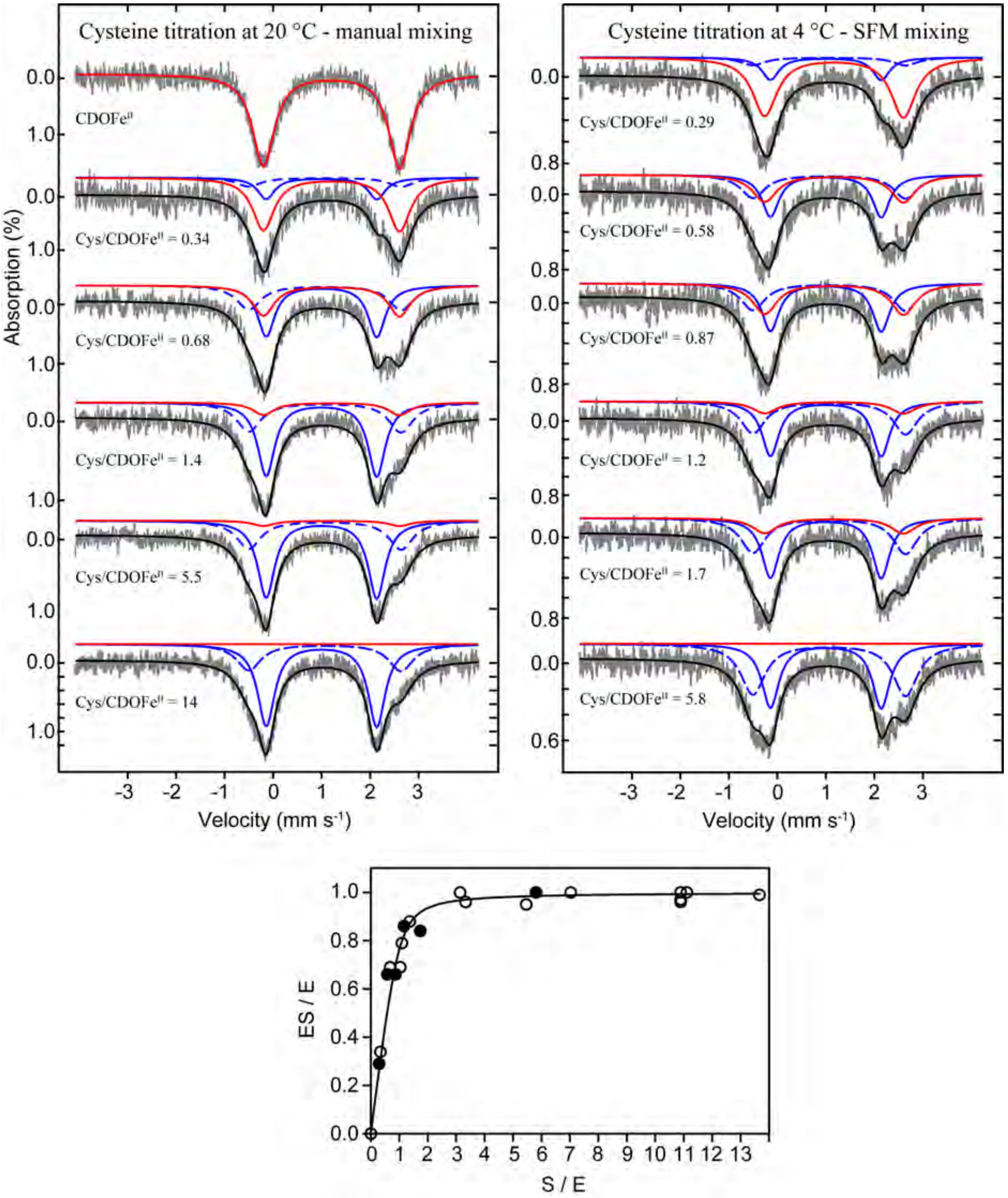

Figure S4. Cysteine binding to CDO monitored by Mössbauer spectra at 20 and 4 °C. The proportion of bound and unbound protein was used and fitted to estimate a lower limit for cysteine binding. Mössbauer data collected under the indicated conditions are shown in the upper two panels as gray traces, and were fitted to three species: CDO:Fe^{II} (red), CDO:Fe^{II}:cysteine site I (solid blue) and CDO:Fe^{II}:cysteine site II (dashed blue). Bottom panel shows a binding curve of complex (as fit in the upper panels) normalized to concentration of Fe(II):CDO. Open circles 20 °C, closed circles 4 °C.

Table S2. Mössbauer parameters of cysteine bound forms of cysteine dioxygenase measured at 5.2 K.

	δ (mm s^{-1})	ΔE_Q (mm s^{-1})	$\Gamma_{L=R}$ (mm s^{-1})[a]
Fe(II):CDO	1.22 ±0.01[b]	2.85 ±0.07	0.60 ±0.04
Fe(II):CDO:cys site I	1.03 ±0.01[c]	2.28 ±0.06	0.43 ±0.05
Fe(II):CDO:cys site II	1.10 ±0.02[c]	3.14 ±0.18	0.61 ±0.08

[a] All spectra were fitted using a Lorentzian lineshape.

[b] Standard deviation represent errors in parameters calculated using data from at least 12 independent experiments.

[c] Standard deviation represent errors in parameters calculated using data from 17 independent experiments.

The presence of two sub-spectra in the Mössbauer spectra is not a result of the presence or absence of the crosslink. Differences in the EPR of inactive oxidized CN bound CDO with and without the crosslink have been previously described.[9] It was therefore initially thought that the presence of two sub-spectra could be caused by the presence of the crosslink. Unfortunately, although we have been able to increase the amount of the crosslink through catalytic turnover,[10] as have others,[9,11] we have been unable to produce 100 % crosslinked protein, especially at the high concentrations (~1 mM) required for Mössbauer spectroscopy. Furthermore, the mechanism of crosslink formation is undefined.[9,11b] Therefore, in order to avoid the unknown artifacts of extensive enzymatic cycling we approached the problem by engineering a C93G substitution within CDO that is unable to form the crosslink.[12] In this recent publication from our laboratory we reported a crystal structure of the C93G CDO variant and provided an extensive and detailed analysis of its enzymatic activity to argue that the absence of crosslink had no structural consequences and that the C93G variant CDO was at least as active as wild type CDO at neutral pH. In this work, we therefore looked at cysteine binding to the active ferrous form of the C93G variant by means of Mössbauer spectroscopy. A cysteine titration of C93G variant CDO clearly shows similar Mössbauer spectra to wild type given above suggesting that the presence of the crosslink is not the cause of this phenomenon (Fig S5). All spectra of anaerobic C93G variant resting state and in complex with cysteine substrate could be reasonably well fitted with the average Mössbauer parameters as determined for wild type CDO thus further illustrating the notion that the two Mössbauer sub-species observed here for cysteine substrate complexes is not a function of presence or absence of crosslink. This is further supported by our structural analysis of C93G.[12] Computational chemistry was therefore used to help and explain the spectra (see below).

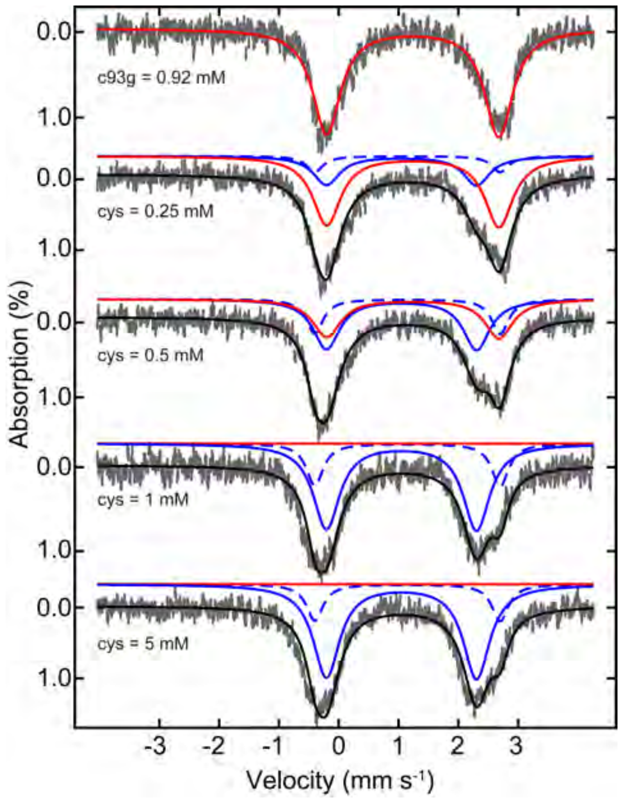

Figure S5. Mössbauer spectra of cysteine binding to C93G variant. All spectra were fitted to average Mössbauer parameters as determined for wild type CDO resting state and cysteine-bound complexes (Table S2). Color scheme as in Fig. S4.

Activity of the cysteine bound complex. To test whether the cysteine bound complex was catalytically active, the samples were exposed to air and the appearance of CSA product monitored by HPLC and MS analysis (Figure S6). The amount of CSA produced was seen to be stoichiometric with respect to the concentration of CDO:Fe^{II}:cysteine complex.

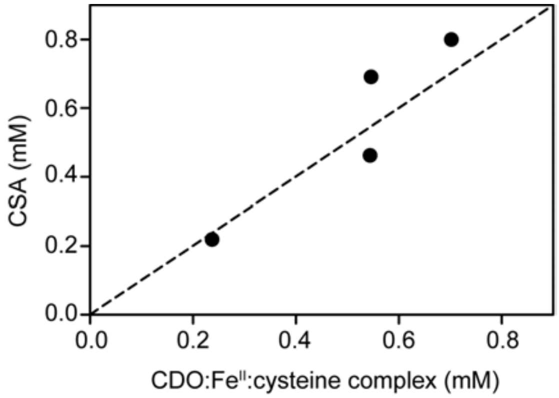

Figure S6. CDO:Fe^{II}:cysteine complex is active and produces stoichiometric amount of CSA. Different concentrations of CDO:Fe^{II}:cysteine complexes were allowed to react with dioxygen and the amount of CSA produced was quantified by HPLC analysis and confirmed by mass spectrometry. Each data point represents the result of an independent titration experiment. Dashed line has a slope of one and illustrates a hypothetical perfect correlation.

8. Kinetic investigation of the short-lived intermediate

The amount of the intermediate observed through stopped-flow was found to depend upon the concentration of the CDO:Fe:Cysteine complex as well as the final concentration of dioxygen added (see Fig S7). Since the intermediate was formed within the dead time of the instrument (1 ms) the rate of formation must be ≥1 × 10^7 $M^{-1}s^{-1}$. Even at the lowest concentration of dioxygen (125 μM) the observed rate must be nearly 2 orders of magnitude higher than the rate of decay (112 ± 5 s^{-1}) and thus ~90% conversion is to be expected. Using this assumption a molar absorptivity of 130 $M^{-1}cm^{-1}$ was estimated but this is ignoring the possibility that dioxygen binding should rather be described as an equilibrium. Thus the true molar absorptivity could be much higher and this represents a lower limit.

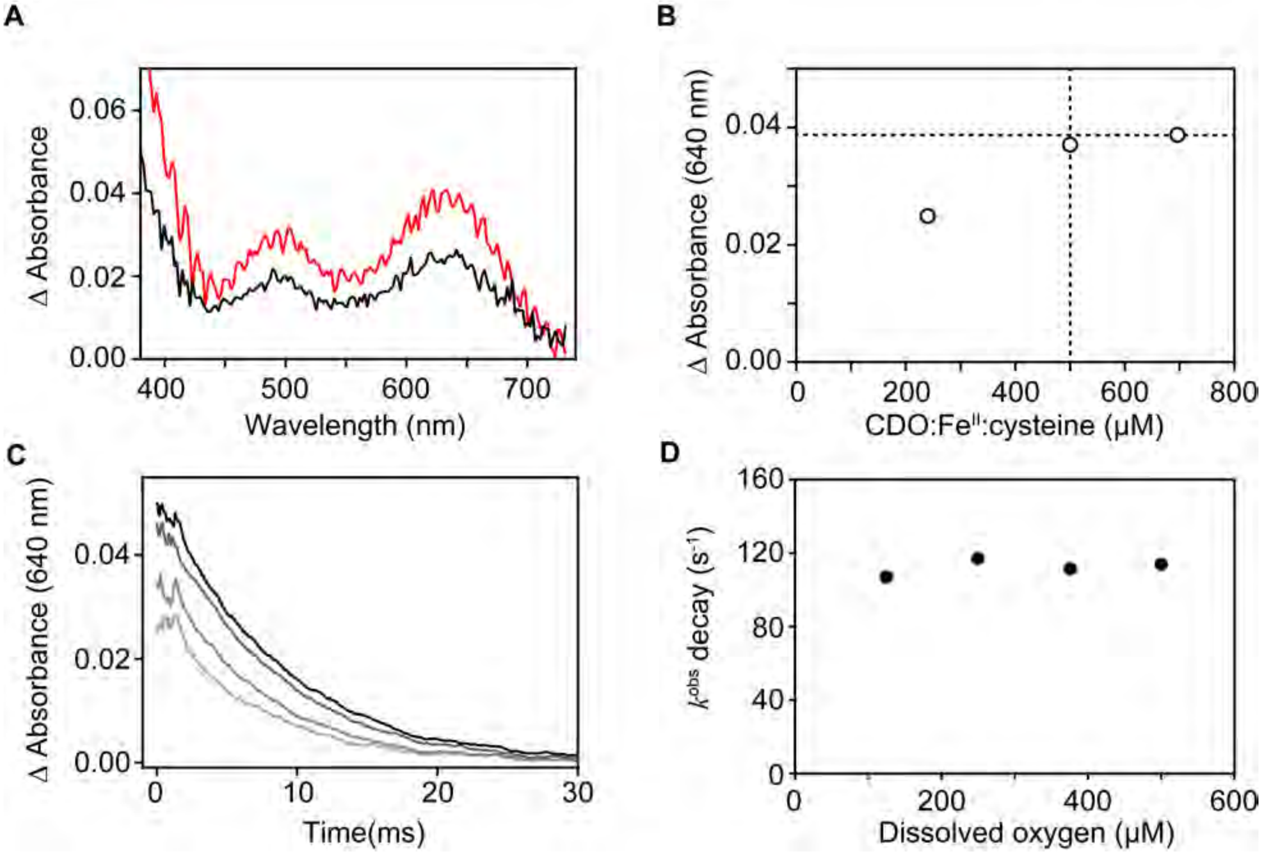

Figure S7. Relationship between the intermediate spectral features and the concentrations of CDO:Fe^{II}:cysteine complex and dissolved oxygen as observed in the Stopped-Flow experiments. (A) Stopped-Flow spectra measured at 1.2 ms after mixing 500 μM (red line) and 240 μM (black line) CDO:Fe^{II}:cysteine with O_2 equilibrated buffer (500 μM). All concentrations are final after 1:1 mixing. (B) The absorbance at 640 nm measured 1.2 ms after mixing as a function of CDO:Fe^{II}:cysteine. The absorbance is seen to plateau once the enzyme substrate concentration is higher than the O_2 concentration. (C) Kinetic traces at 640 nm obtained by mixing 500 μM CDO:Fe^{II}:cysteine with various concentrations of dissolved oxygen: 500 μM (black line), 376 μM, 250 μM and 125 μM illustrated by the lines with lighter shades of gray. (D) Variation of rate of decay as a function of O_2. Each point is the average of at least two measurements.

Due to the inhomogeneous nature of WT CDO possibly interfering with our kinetic study we also carried out comparable stopped-flow studies on our C93G variant. It was found that again formation of the intermediate was observed within the mixing time of the experiment but now decay was even faster with a rate of 580 ± 30 s^{-1} that is near the limit of detection (Figure S8). This meant that this avenue of research was not pursued any further but it does strongly suggest that the uncrosslinked cysteine 93 in WT CDO interferes with the reaction, causing a difference in the rate of reaction of crosslinked and uncrosslinked protein. Indeed, in our previous study we have shown that although there is a shift in the pH at which the maximum rate is observed, the overall kinetic activity of 100% crosslinked WT CDO and C93G are quite comparable. This would also explain the lower activity of the C93A and C93S variants compared to our C93G variant.

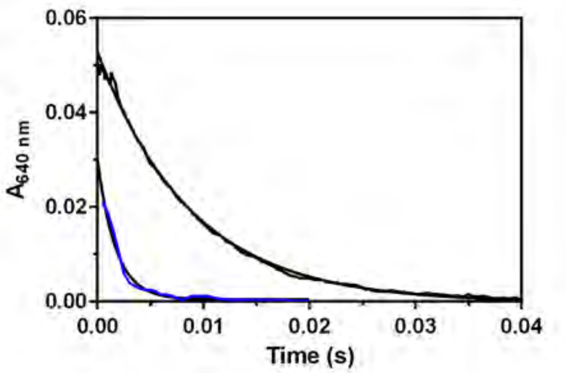

Figure S8. Stopped-flow comparison of the decay of the intermediate followed at 640 nm produced by the addition of dissolved dioxygen with cysteine bound complexes of WT CDO (black) and C93G (blue).

9. Freeze-quench study of single turnover

The ability to form a stable and tight cysteine bound CDO complex enabled single turnover of the enzyme to be explored. Anaerobic mixing of cysteine bound complex with deoxygenated buffer and freezing after 20 ms led to the cysteine bound complex being recovered (middle spectrum, Fig S9A). In contrast, when the same cysteine-bound complex was mixed with one equivalent of dioxygen dissolved in buffer and frozen at 20 ms, resting state CDO was obtained. Importantly, when the sample was analyzed by HPLC-ELSD, one equivalent of CSA product was found to have formed. Thus, the dioxygenation step is extremely fast (≤ 20 ms) which corresponds to a rate of ≥ $3 \times 10^5\ M^{-1}\ s^{-1}$.

Freeze quench was repeated at Penn State by B. Zhang and C. Pollock with freezing times down to 10 ms using both WT CDO and C93G (Fig S9B). The Mössbauer spectra measured at 4.2 K at zero field show again that the enzyme remains in the ferrous resting state.

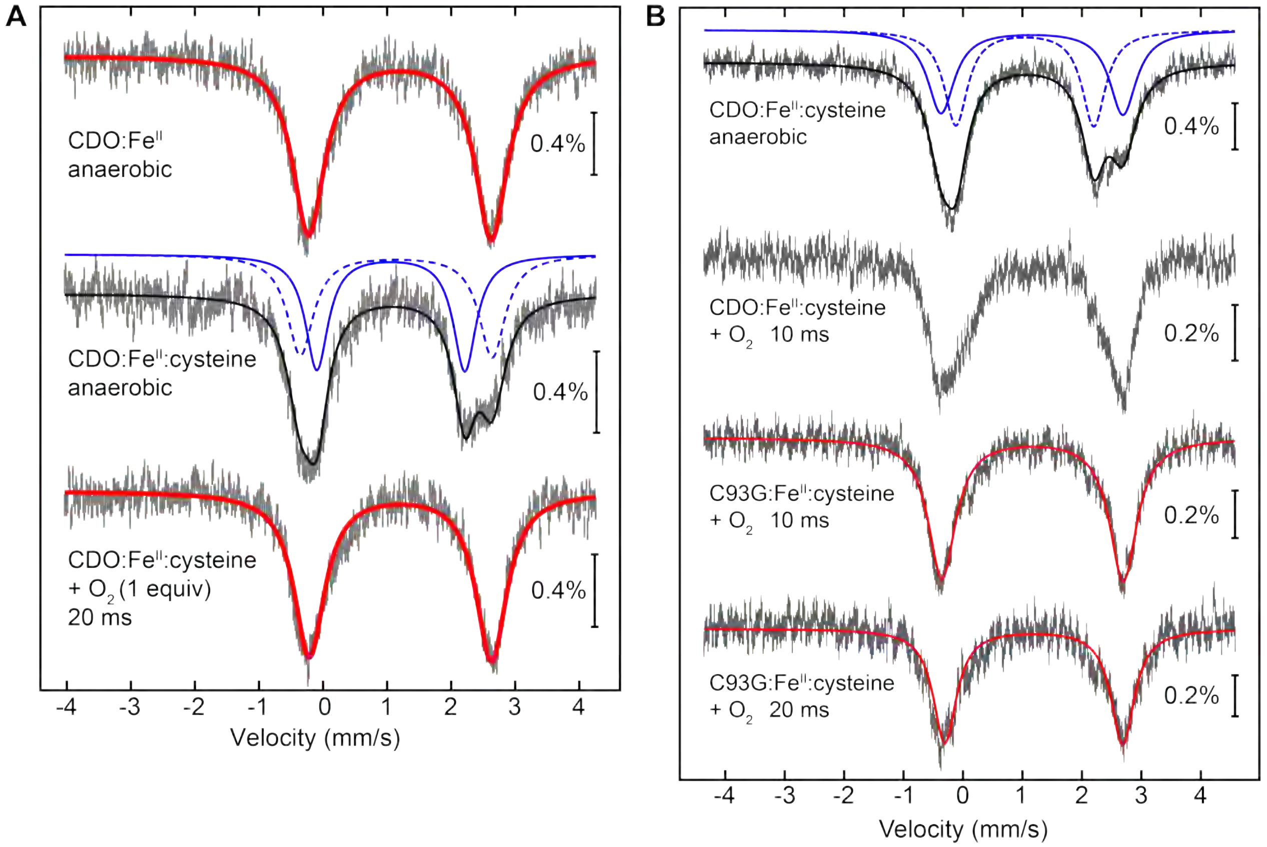

Figure S9. A) Mössbauer spectra of resting state CDO, cysteine bound CDO mixed with degassed buffer and frozen after 20 ms, and cysteine bound CDO after addition of dissolved O_2 and frozen after 20 ms. Spectra measured with 47 mT magnetic field applied parallel to the γ-rays. The sample has returned to resting state within 20 ms with concomitant production of CSA (652 μM CDO:Fe^{II}:cysteine produced 573 μM CSA, 88% conversion). B) Mössbauer spectra of anaerobic cysteine bound control, sample quenched 10 ms after addition of O_2, cysteine bound C93G variant frozen 10 and 20 ms after addition of dissolved O_2. Spectra measured in absence of magnetic field. Color scheme as is Fig. S4.

10. Computational methods

The calculations started off from the chemical structures of the catalytic cycle reported previously.[13] However, for consistency all structures were reoptimized using the *Jaguar* 7.9 software package.[14] A minimum level of constraints was included in the model to enable fidelity in the structural representation of the active region. This region consisted of the iron bound to the Cys substrate with imidazole groups representing histidine residues: His_{86}, His_{88}, His_{140} and His_{155}. Also included was a methylguanidinium group to represent Arg_{60}, a phenolate group in place of Tyr_{157} and methylsulfide for Cys_{93} to give a total atom count of 112 for the model. In order to prevent the model from undergoing too many structural changes with respect to the crystal structure we constraint one atom and the plane of symmetry of each of the second sphere amino acid residues. Thus, the phenol oxygen atom of Tyr_{157}, the carbon atom of the methyl group of Arg_{60} and one of the nitrogen atoms of His_{155} were fixed in position with respect to the iron-histidine center. Scheme S1 below gives the general mechanism of the proposed catalytic cycle of CDO enzymes. The Scheme starts from the pentacoordinated resting state structure (**A'**) that can convert to a hexacoordinated structure by binding of water (structure **A**). Upon binding of molecular oxygen the water molecule is displaced and an iron(III)-superoxo structure is formed (**B**). The terminal oxygen atom of the iron(III)-superoxo group attacks the sulfur of the cysteinate group to form a ring-structure (**C**), which can homogeneously split into a sulfoxide and iron(IV)-oxo species (**D**). Note the sulfoxide-bound complex (**D**) rotates from sulfur-bound to oxygen-bound (**D'**) to the iron center. The latter transfers the oxygen atom from the iron(IV)-oxo group to substrate in a final step to form cysteine sulfinic acid product complex (**E**). We calculated the absorption spectra of all intermediates in all low lying spin states (singlet, triplet and quintet) using *Gaussian*-09.[15]

Geometry optimizations were carried out on the quintet and triplet ground state for each structure using the unrestricted hybrid density functional method B3LYP,[16] with a basis set containing 6-31G+ double-ζ basis set on all atoms except for the iron were a double-ζ quality LACVP basis set that includes an effective core potential was used (basis set BS1).[17] Additional calculations were done with the triple-ζ quality LACV3P+ basis set on iron (with core potential) and 6-311+G* on the rest of the atoms (basis set BS2). To test the consistency of the results, we also ran single point calculations with the PBE0 and BP86 methods,[18] but these studies gave similar results on spin state ordering and relative energies. To test the effects of the environment in the enzyme we ran single point calculations on the optimized geometries using the self–consistent reaction field procedure with a dielectric constant of $\varepsilon = 5.7$ and a probe radius of 2.7 Å.

Vibrational frequencies were calculated on the optimized structures using the *Gaussian*-09 software package and free energies were calculated at 298.15 K and 1 atm, which resulted in local minima with real frequencies only. To establish the absorption spectra, we then ran time-dependent density functional theory (TD-DFT) calculations on all systems using *Gaussian*-09.

Mössbauer parameters were calculated for each B3LYP/BS1 optimized geometry of the catalytic cycle with the *Orca* 2.9v program package.[19] These calculations use approaches discussed before with the unrestricted B3LYP employed with basis set CP(PPP) with effective core potential on iron and TZVP on the rest of the atoms. The electron density (ρ_0)Fe and quadrupole splitting (ΔE_Q) thus obtained were then fitted to the previously determined parameters[20] to obtain the isomer shift (δ).

To investigate the origin of the two species observed in the Mössbauer spectra of cysteine-bound CDO we predicted Mössbauer parameters computationally. Initially the iron bound protein complex was calculated, as only a single species is present (Table S3). Prediction of Mössbauer parameters is highly dependent upon slight changes in coordination number and geometry.[21] A truncated first coordination sphere alone (R_1) under-estimated the isomer shift quite considerably and this was improved by extension of the coordinating ligands (R_2). Addition of the H-bonding network suggested by the crystal structures[22] and previous calculations improved the isomer shift but in all calculations the quadrupole splitting is over-estimated by ~0.9 mm s^{-1}. Thus the second coordination sphere was included in calculations of the cysteine-bound *ES* complex. Energetically, the triplet state lies so high in energy that only quintet states were considered (see supporting information). It

can be seen that the data can be best explained by a complex *with* (A_3) and *without* (A'_3) water in the sixth coordination site (Table S3).

To further calibrate the spectroscopic calculations, we decided to run a series of CASSCF and NEVPT2 calculations on the iron(III)-superoxo and bicyclic ring structures in the quintet spin state. These studies use truncated versions of the optimized DFT models, which only include the first coordination sphere of residues around the iron center (see Scheme S2) All calculations were run using the ORCA 2.9 software package[19] and the basis set TZVP. Using CASSCF/NEVPT2 we calculated the quintet ground states of the iron(III)-superoxo and bicyclic ring structures using an active space containing 10 orbitals and 14 electrons. We made sure to include all the high energy, antibonding orbitals on the iron as well as the high energy orbitals associated with the dioxygen molecules and sulfur (Figure S26). We then searched for the first 150 excitation associated with these two species.

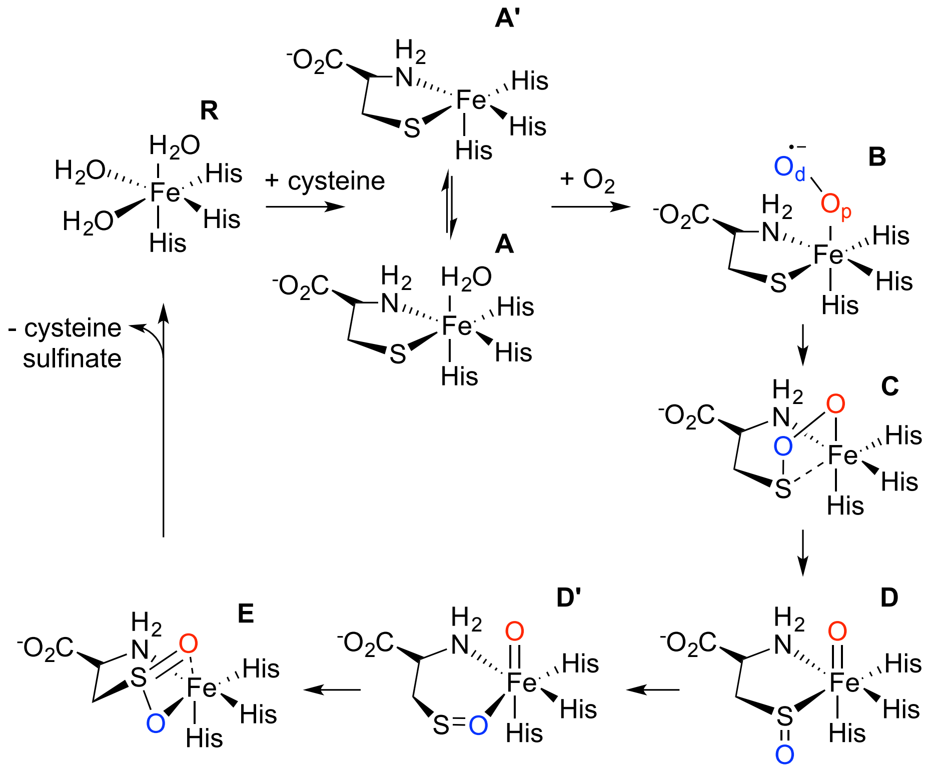

Scheme S1: Catalytic cycle of CDO enzymes with labelling of the individual oxygen bound and resting state structures.

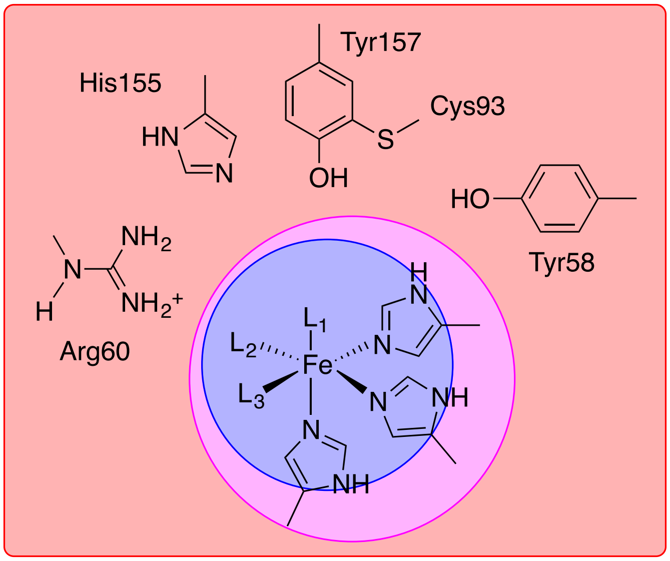

X_1 = Fe(Im)$_3$($L_1L_2L_3$) (Im = imidazole)
X_2 = Fe(His)$_3$($L_1L_2L_3$)
X_3 = X_1 · Arg60 : His155: Cys93-Tyr157: Tyr58

Scheme S2: Description of the 1st and 2nd coordination sphere used to calculate Mössbauer parameters. The labeling for R_1 and R_2 is similar to that of X_1 and X_2, but $L_1 = L_2 = L_3 = H_2O$.

Table S3. Experimental and calculated Mössbauer parameters.

	δ (mm s^{-1})	ΔE_Q (mm s^{-1})
CDO:Fe^{II} (expt)	1.22	2.85
R_1	0.89	3.60
R_2	1.06	3.71
CDO:Fe^{II}:cys I (expt)	1.03	2.28
CDO:Fe^{II}:cys II (expt)	1.10	3.14
$^5A_3$	0.98	3.85
$^5A'_3$	0.87	4.26
^5B	0.91	3.82
^5C	0.92	3.39
^3D	0.31	1.84
^5E	1.00	3.43

Table S4. Absolute values for electronic energy with zero-point correction, free energy, and solvent correction obtained at UB3LYP/BS2//UB3LYP/BS1 level of theory. All structures performed on model X_3.

	E1 (au) BS1	ZPE (au) BS1	E+ZPE (au) BS1	G (au) BS1	E2 (au) BS2	Esolv (au) BS2
^{5}A'	-3282.0324	0.9166	-3281.1157	-3281.2287	-3283.3604	-3283.4617
^{3}A'	-3282.0201	0.9185	-3281.1016	-3281.2184	-3283.3475	-3283.4480
^{5}A	-3358.4509	0.9412	-3357.5097	-3357.6295	-3359.8247	-3359.9235
^{3}A	-3358.4306	0.9433	-3357.4873	-3357.6048	-3359.8037	-3359.9004
^{3}R	-1148.7003	0.3815	-1148.3187	-1148.3806	-1149.2550	-1149.4855
^{5}R	-1148.7312	0.3805	-1148.3507	-1148.4157	-1149.2900	-1149.5189

Table S5. Relative values for electronic energy with zero-point correction, free energy, and solvent correction obtained at UB3LYP/BS2//UB3LYP/BS1 level of theory. All structures performed on model X_3.

	ΔE BS1	ΔE+ZPE BS1	ΔG BS1	ΔE BS2	ΔE+ZPE BS2	ΔG BS2	ΔEs+ZPE BS2	ΔGs BS2
^{5}A'	0.00	0.00	0.00	0.00	0.00	0.00	0.00	0.00
^{3}A'	7.71	8.88	6.41	8.09	9.27	6.79	9.81	7.33
^{5}A	0.00	0.00	0.00	0.00	0.00	0.00	0.00	0.00
^{3}A	12.74	14.09	15.47	13.19	14.54	15.92	15.84	17.22
^{5}R	0.00	0.00	0.00	0.00	0.00	0.00	0.00	0.00
^{3}R	19.43	20.06	22.05	21.95	22.58	24.57	21.58	23.57

Table S6. Relative values for electronic energy with zero-point correction, free energy, and solvent correction obtained at UBP86/BS2//UB3LYP/BS1 level of theory. All structures performed on model X_3.

	E2 (au) BS2	Esolv BS2	ΔE BS2	ΔE+ZPE BS2	ΔG BS2	ΔEs+ZPE BS2	ΔGs BS2
^{5}A	-3359.9366	-3360.0340	0.00	0.00	0.00	0.00	0.00
^{3}A	-3359.9344	-3360.0293	1.43	2.78	4.16	4.27	5.64
^{5}R	-1149.3249	-1149.5546	0.00	0.00	0.00	0.00	0.00
^{3}R	-1149.3466	-1149.5746	13.62	14.25	16.24	13.17	15.16

Table S7. Group mulliken spin densities obtained at UB3LYP/BS1. All structures performed on model X_3.

	Fe	Lig(3His)	Sub(Cys)	Others
^{3}A'	2.03	0.00	-0.04	0.00
^{5}A'	3.72	0.12	0.15	0.00
	FeOO~	Lig(3His)	Sub(Cys)~	Others
^{5}C	3.82	0.09	0.06	0.03
	FeO	Lig(3His)	Sub(Cys)-O	Others
^{3}D	2.74	-0.03	-0.74	0.04
	FeO~	Lig(3His)	Sub(Cys)SO	Others
^{5}E	3.82	0.08	0.07	0.03
	FeOO	Lig(3His)	Sub(Cys)	Others
^{5}B	3.29	0.09	0.59	0.03
	Fe(H2O)	Lig(3His)	Sub(Cys)	Others
^{3}A	2.03	0.00	-0.03	-0.01
^{5}A	3.78	0.08	0.12	0.03
	Fe(3H2O)	Lig(3His)		
^{3}R	1.98	0.02		
^{5}R	3.86	0.14		

Table S8. Group mulliken charges obtained at UB3LYP/BS1. All structures performed on model X_3.

	Fe	Lig(3His)	Sub(Cys)	Others
^{3}A'	0.39	0.47	-0.69	0.83
^{5}A'	0.60	0.41	-0.86	0.85
	FeOO~	Lig(3His)	Sub(Cys)~	Others
^{5}C	-0.12	1.19	-0.16	0.09
	FeO	Lig(3His)	Sub(Cys)-O	Others
^{3}D	-0.09	1.25	-0.34	0.18
	FeO~	Lig(3His)	Sub(Cys)SO	Others
^{5}E	0.00	1.13	-0.24	0.11
	FeOO	Lig(3His)	Sub(Cys)	Others
^{5}B	0.27	1.22	-0.55	0.07
	Fe(H2O)	Lig(3His)	Sub(Cys)	Others
^{3}A	0.40	1.03	-0.73	0.14
^{5}A	0.65	0.97	-0.93	0.15
	Fe(3H2O)	Lig(3His)		
^{3}R	1.24	0.76		
^{5}R	1.32	0.68		

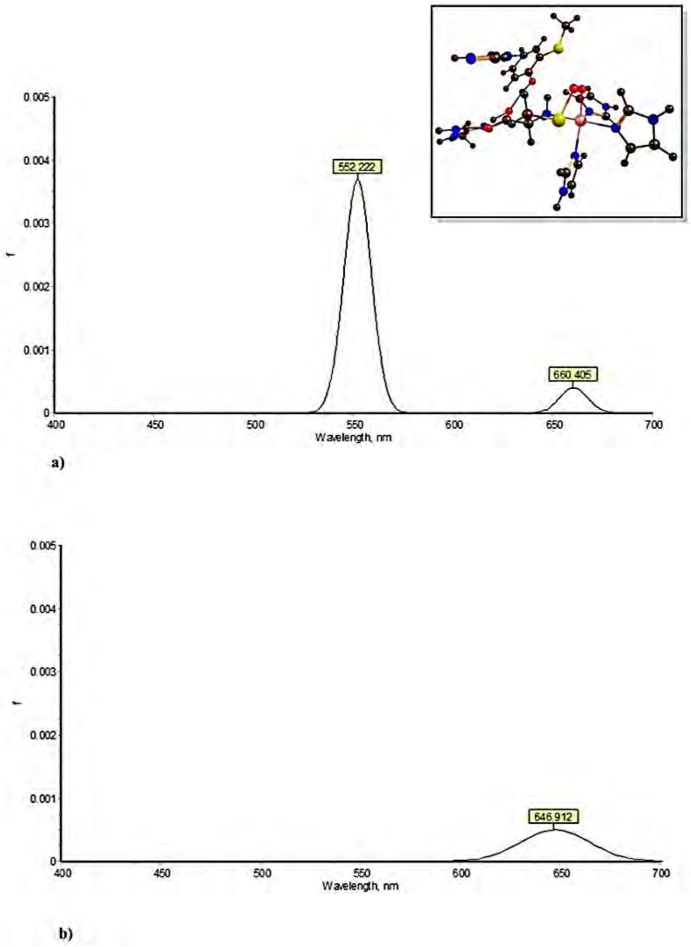

Figure S10. TD-DFT calculated absorption spectra of ^{5,3}C at UB3LYP/BS1 in Gaussian. a) quintet spin state. b) triplet spin state.

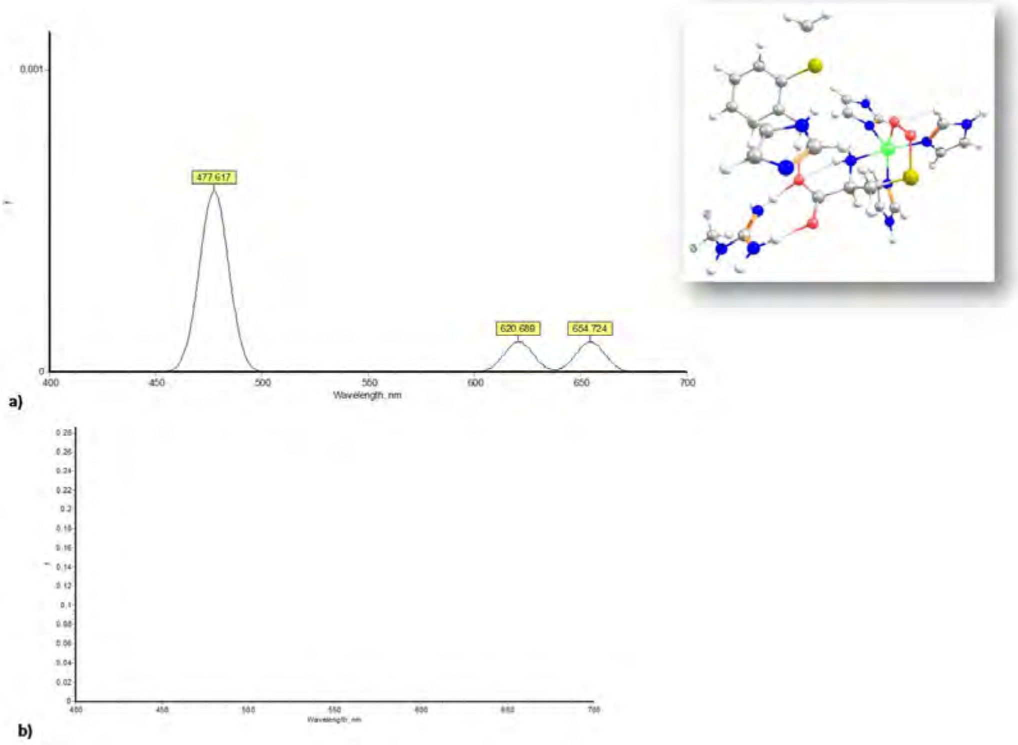

Figure S11. TD-DFT calculated absorption spectra of ^{5,3}C at UB3LYP/BS2 in Gaussian. a) quintet spin state. b) triplet spin state.

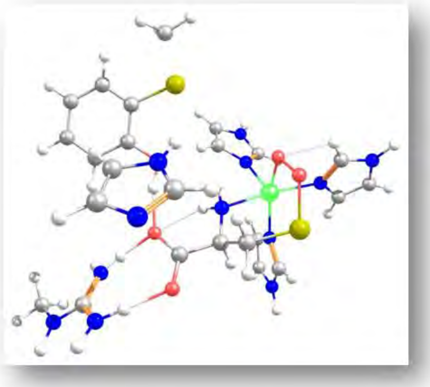

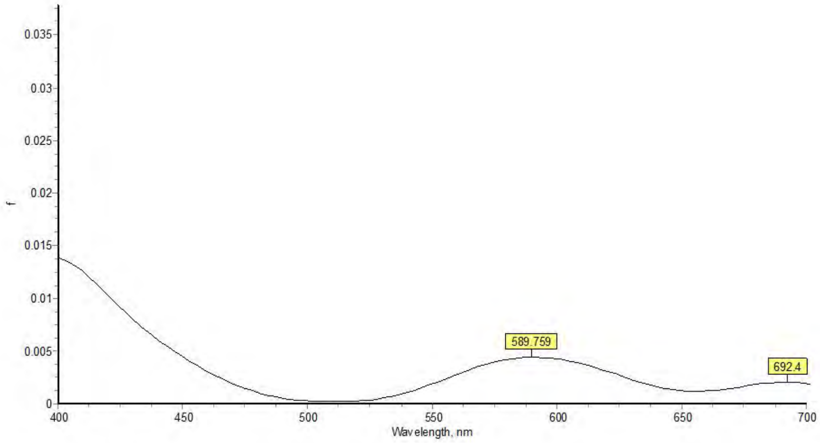

Figure S12. TD-DFT calculated absorption spectra of ^{3}C at UPBE0/BS2 in Gaussian.

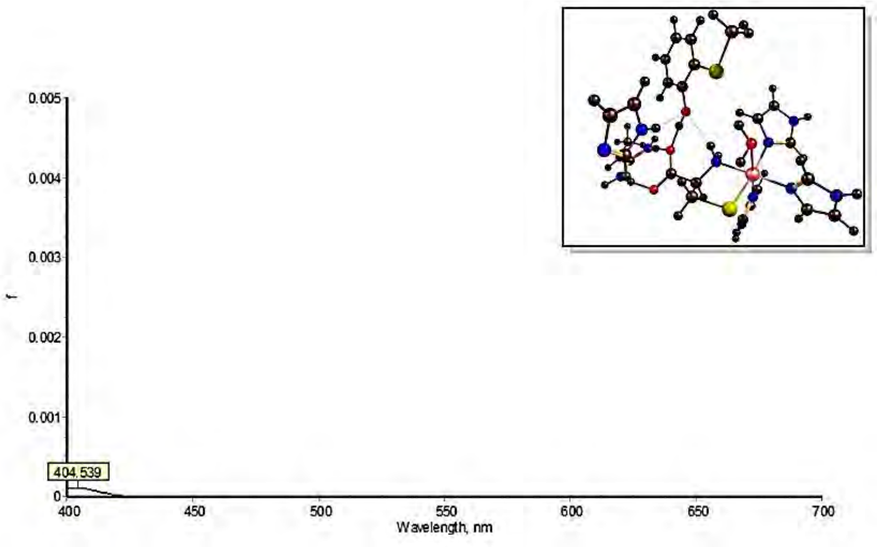

a)

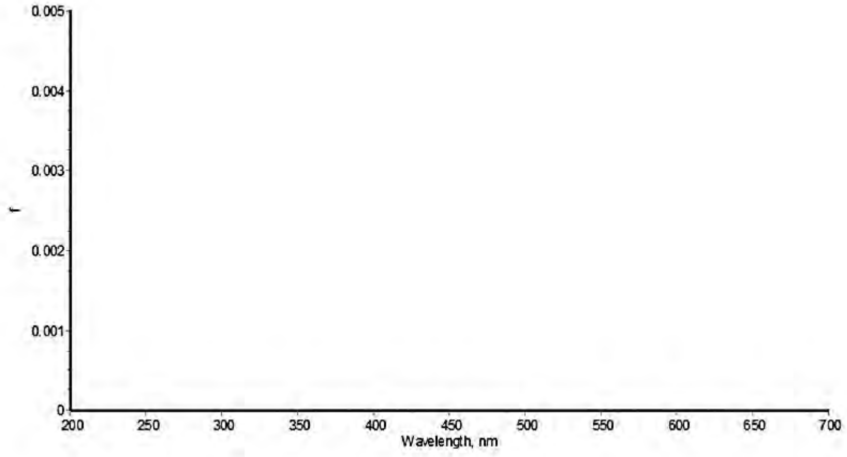

b)

Figure S13. TD-DFT calculated absorption spectra of ^{5,3}A at UB3LYP/BS1 in Gaussian. a) quintet spin state. b) triplet spin state.

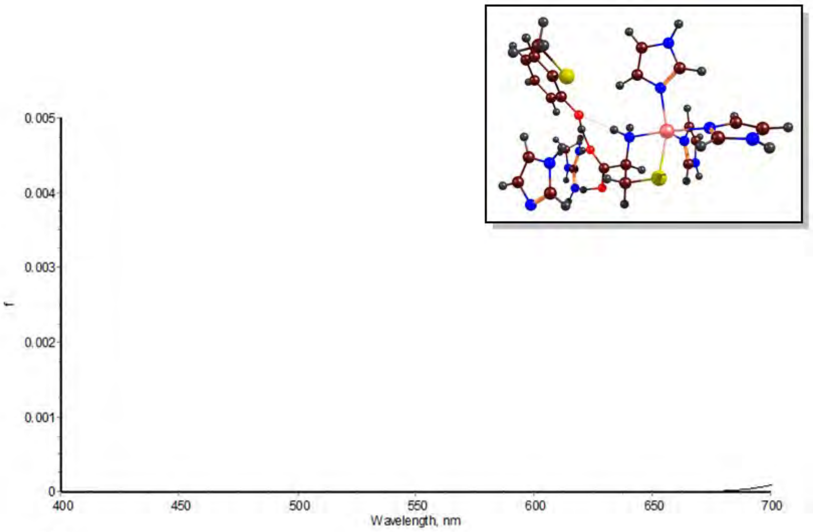

a)

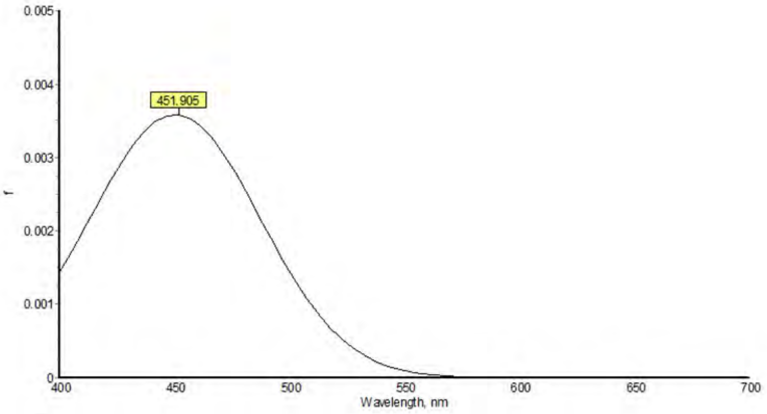

b)

Figure S14. TD-DFT calculated absorption spectra of ^{5,3}A' at UB3LYP/BS1 in Gaussian. a) quintet spin state. b) triplet spin state.

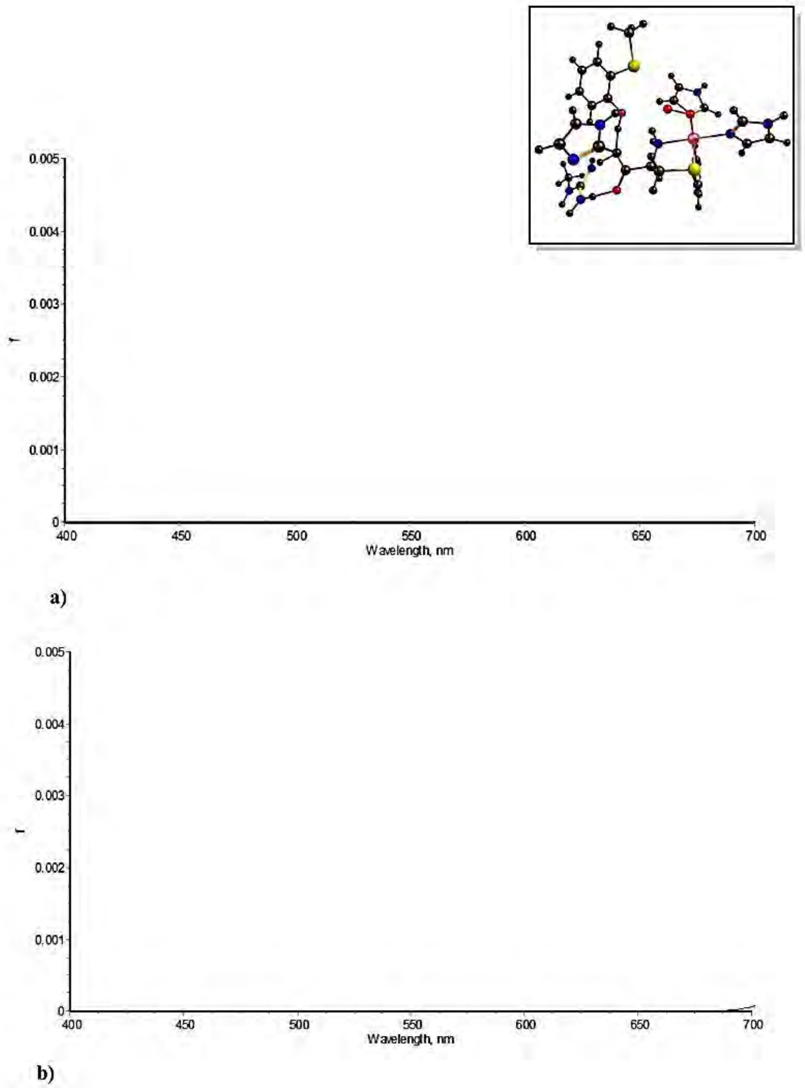

Figure S15. TD-DFT calculated absorption spectra of ^{5,3}B at UB3LYP/BS1 in Gaussian. a) quintet spin state. b) triplet spin state.

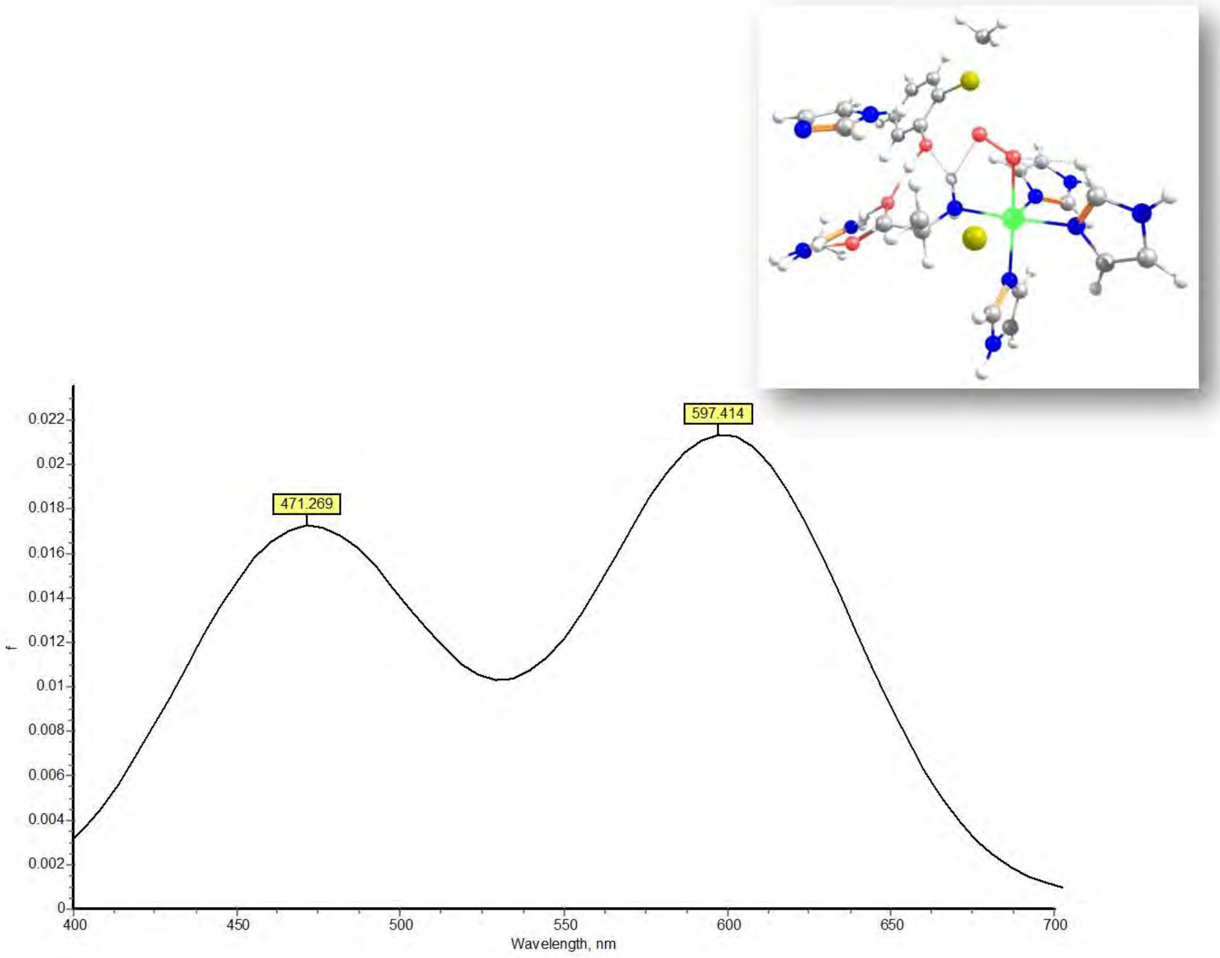

Figure S16. TD-DFT calculated absorption spectra of ^{5}B at UB3LYP/BS2 in Gaussian.

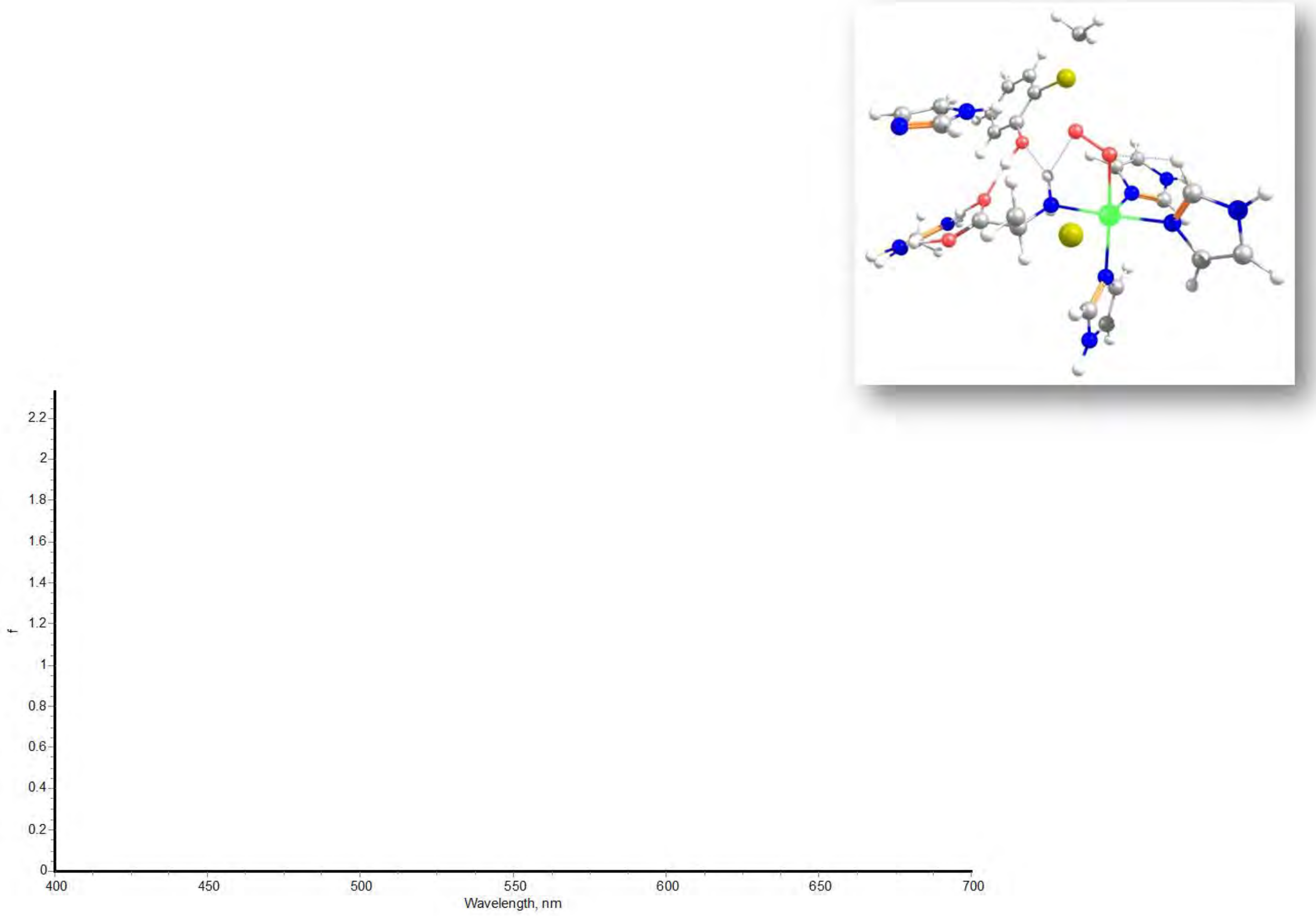

Figure S17. TD-DFT calculated absorption spectra of ^{3}B at UBP86/BS2 in Gaussian.

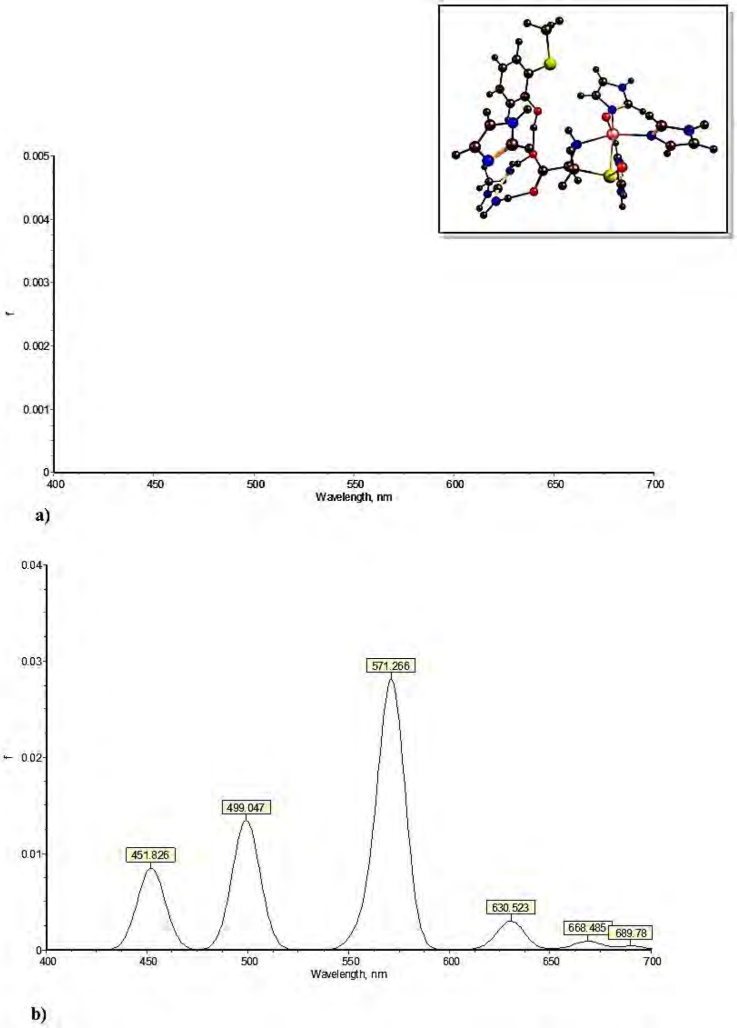

Figure S18. TD-DFT calculated absorption spectra of ^{5,3}D at UB3LYP/BS1 in Gaussian. a) quintet spin state. b) triplet spin state.

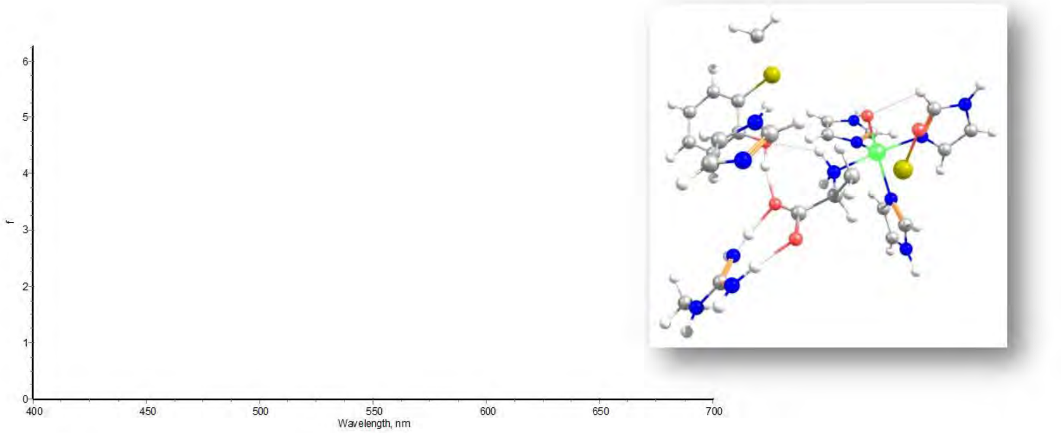

a)

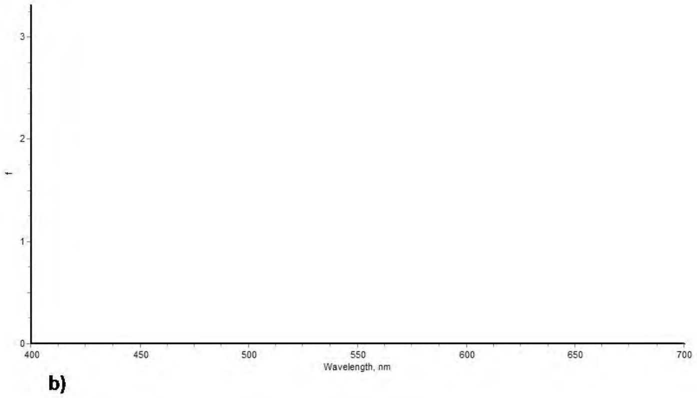

b)

Figure S19. TD-DFT calculated absorption spectra of ^{5,3}D at UB3LYP/BS2 in Gaussian. a) quintet spin state. b) triplet spin state.

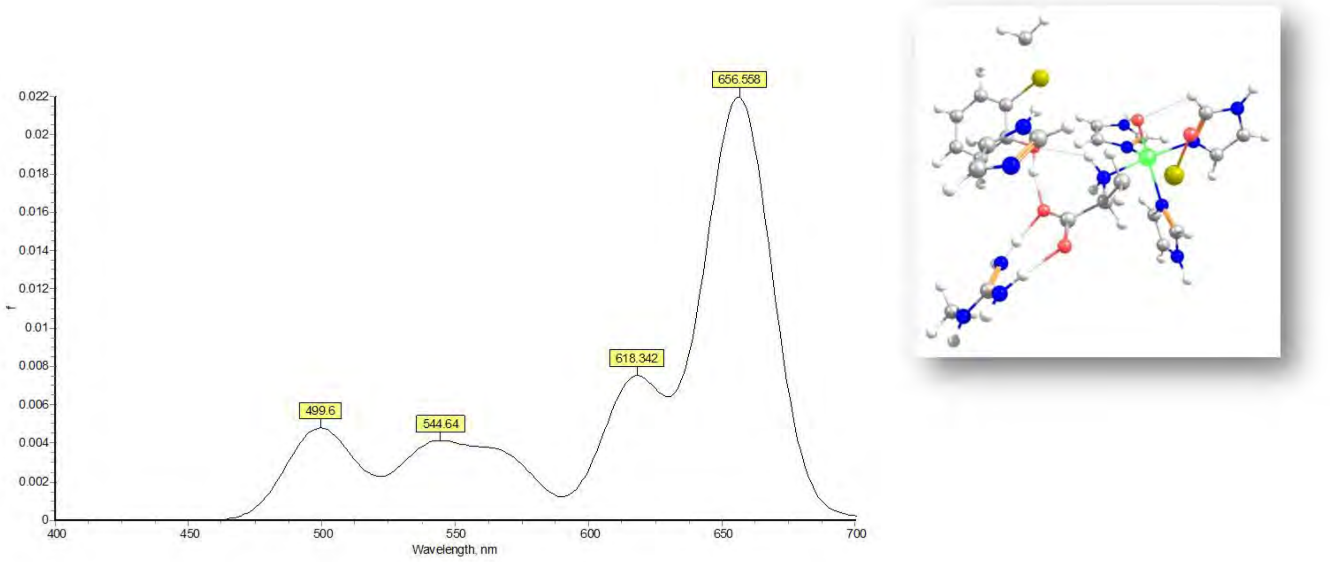

a)

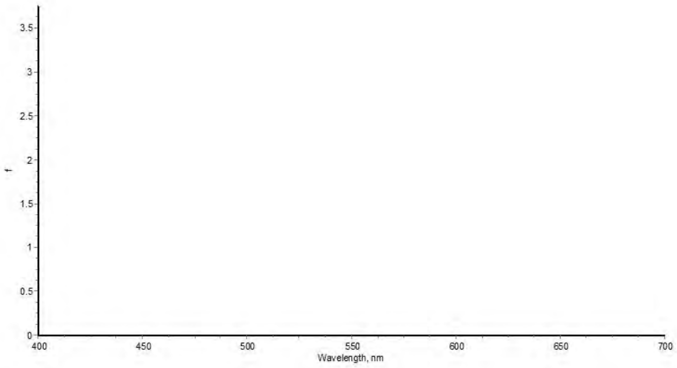

b)

Figure S20. TD-DFT calculated absorption spectra of ^{5,3}D at UBP86/BS2 in Gaussian. a) quintet spin state. b) triplet spin state.

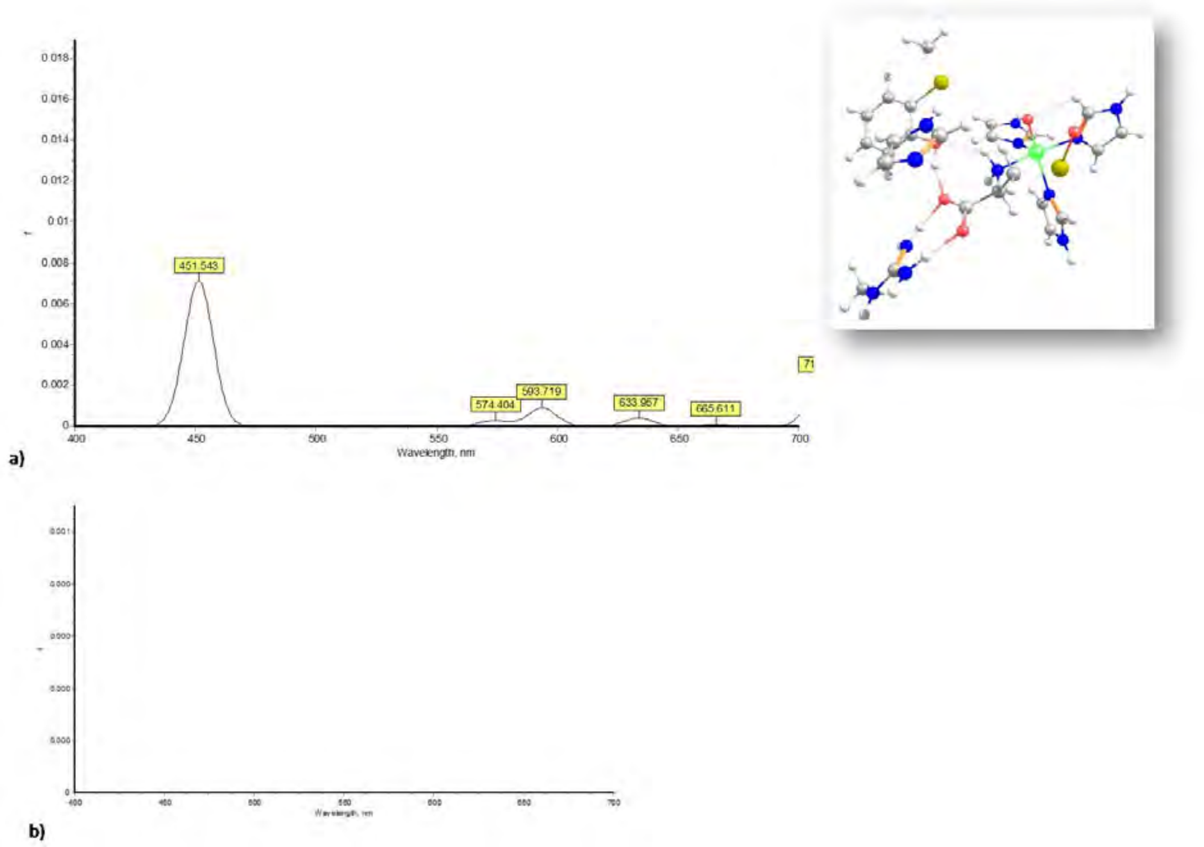

Figure S21. TD-DFT calculated absorption spectra of ^{5,3}D at UPBE0/BS2 in Gaussian. a) quintet spin state. b) triplet spin state.

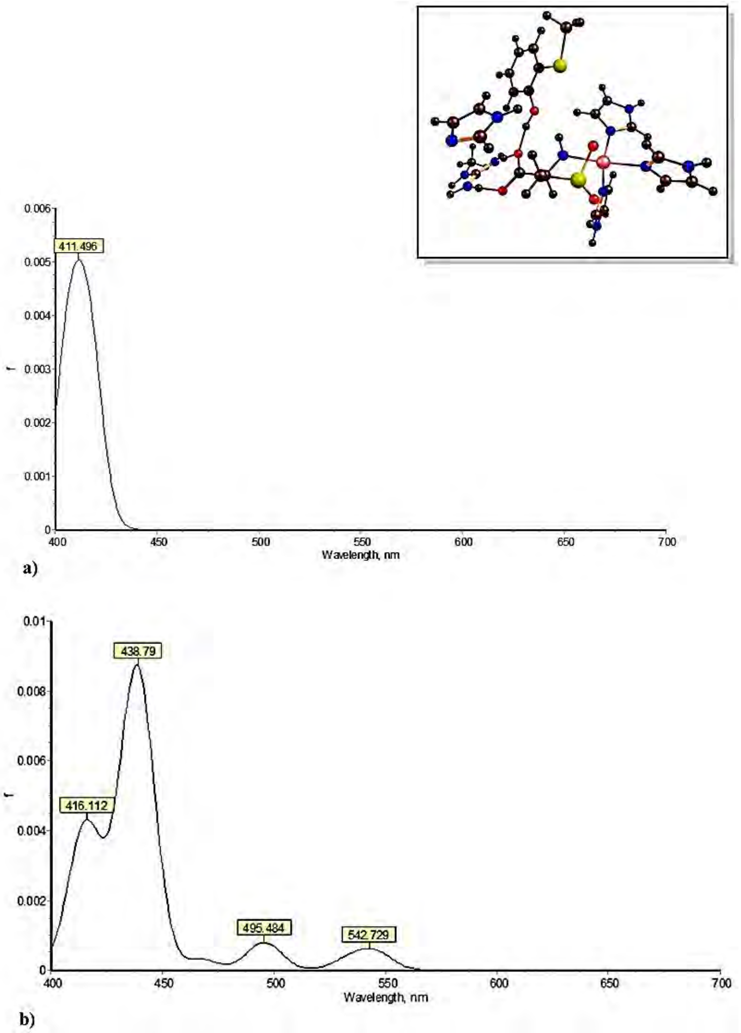

Figure S22. TD-DFT calculated absorption spectra of ^{5,3}E at UB3LYP/BS1 in Gaussian. a) quintet spin state. b) triplet spin state.

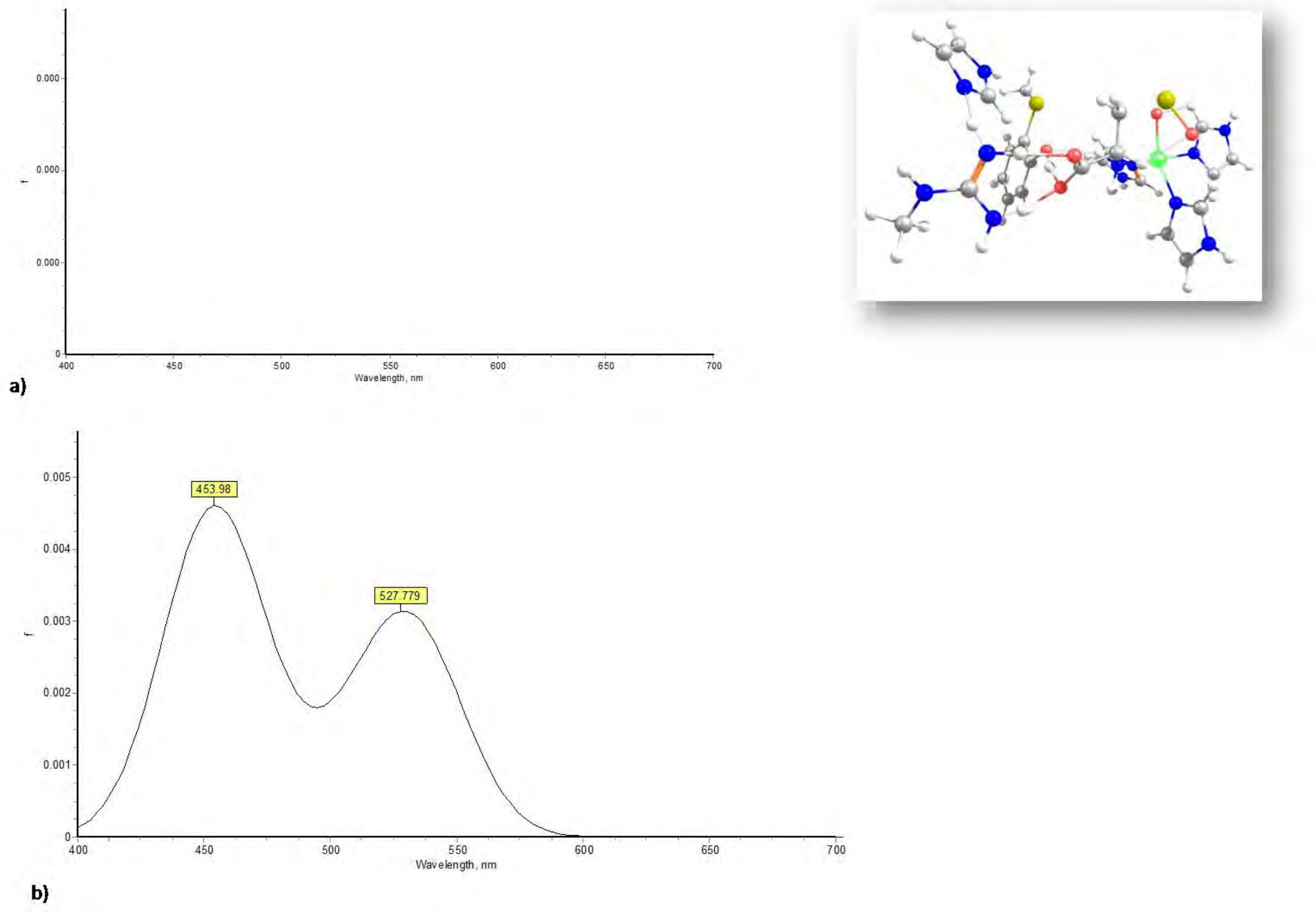

Figure S23. TD-DFT calculated absorption spectra of ^{5,3}E at UPBE0/BS2 in Gaussian. a) quintet spin state. b) triplet spin state.

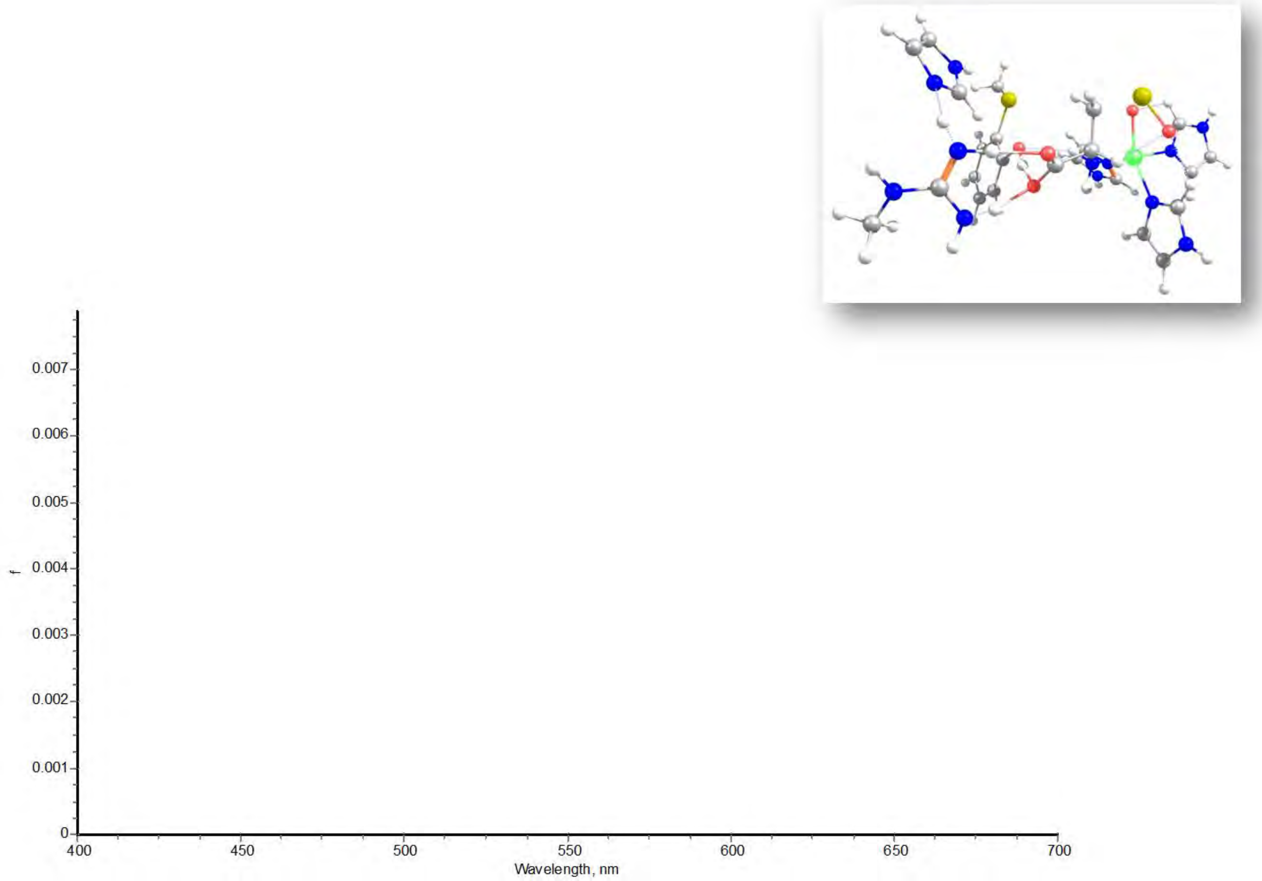

Figure S24. TD-DFT calculated absorption spectra of ^{5}E at UB3LYP/BS2 in Gaussian.

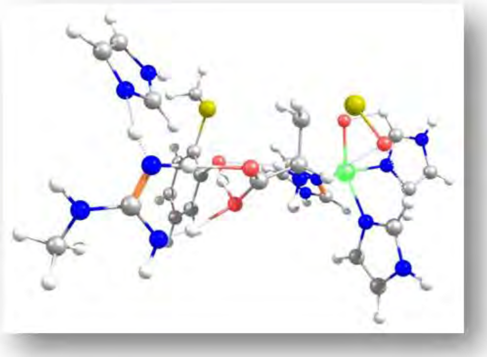

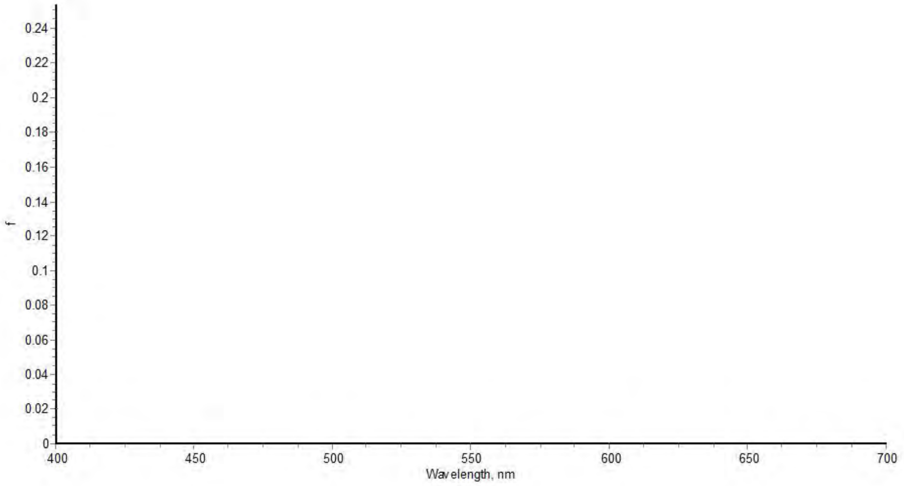

Figure S25. TD-DFT calculated absorption spectra of ^{3}E at UBP86/BS2 in Gaussian.

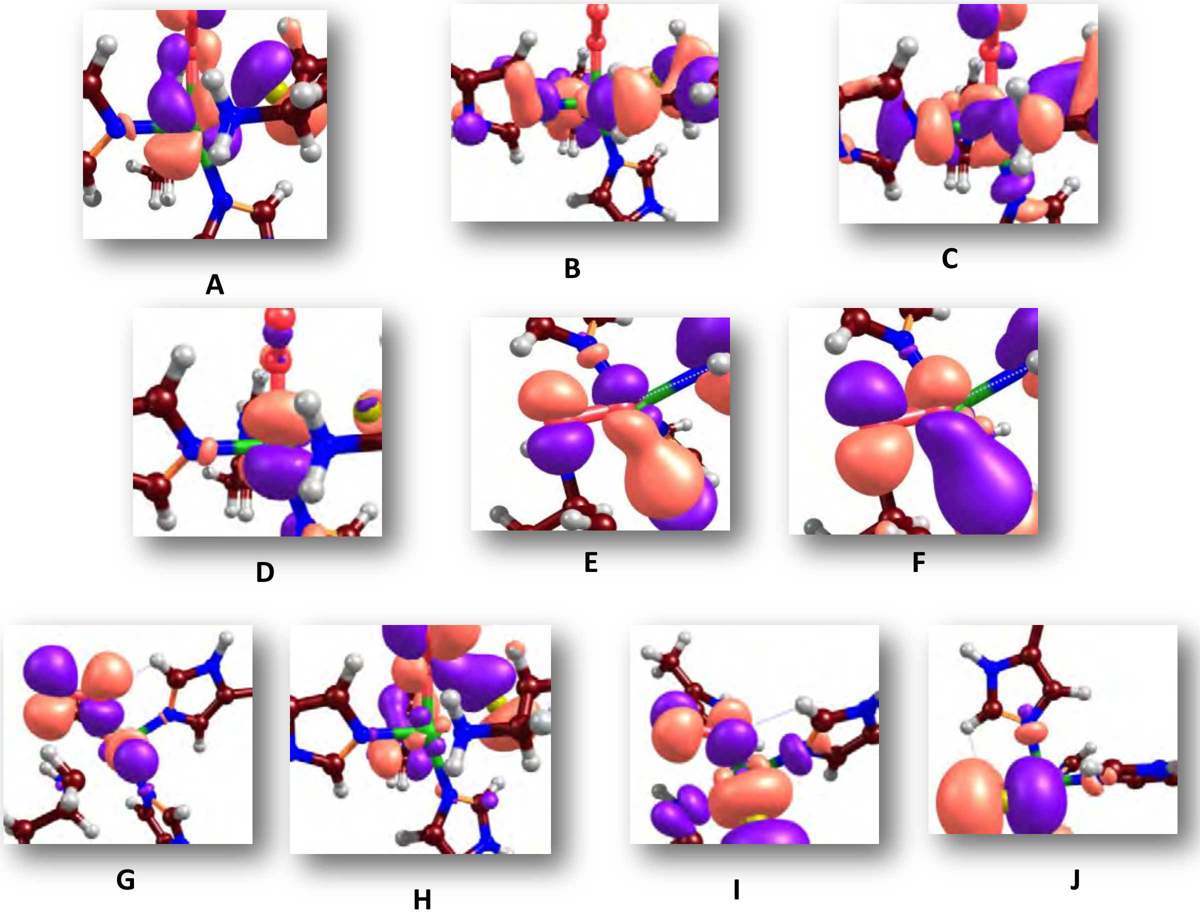

Figure S26. CASSCF(10,14)/BS2 orbitals.

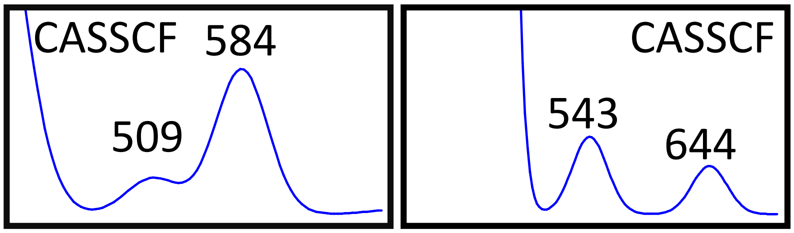

Figure S27. CASSCF(10,14)/BS2 calculated absorption spectra of the iron(III)-superoxo (left) and iron(III)-bicyclic ring structures (right).

Cartesian Coordinates

$^{3}A'_{3}$

Fe	-0.11351	0.18197	0.217804
N	-0.02669	0.111903	2.474717
C	1.071163	0.565076	3.086566
N	0.975587	0.349783	4.43558
C	-0.24225	-0.27518	4.709711
C	-0.84787	-0.4157	3.479802
H	1.916227	1.010859	2.588369
H	1.672076	0.609417	5.117008
N	-2.04929	-0.4758	-0.03348
C	-2.45534	-1.18807	-1.08655
N	-3.81174	-1.34526	-1.04152
C	-3.19652	-0.16944	0.710691
C	-4.30662	-0.70578	0.097414
C	-5.75432	-0.68836	0.459535
H	-6.36468	-0.22097	-0.32288
H	-5.90371	-0.11806	1.379767
H	-3.14197	0.39275	1.62723
H	-1.82662	-1.56041	-1.87403
H	-4.36633	-1.81122	-1.7431
H	-6.1463	-1.69882	0.628897
N	0.588144	-1.68489	-0.04082
C	1.586677	-2.0477	-0.85379
N	1.824601	-3.38575	-0.71301
C	0.942983	-3.90979	0.236611
C	0.179969	-2.8378	0.639656
C	0.941639	-5.34611	0.642357
H	1.902031	-5.64802	1.078246
H	0.73685	-6.01233	-0.20497
H	-0.60663	-2.8062	1.371994
H	2.140361	-1.38551	-1.49692
H	2.537133	-3.90353	-1.2036
H	0.168918	-5.52172	1.395105
N	-0.7679	2.118539	0.127727
C	0.266537	3.135987	0.511601
C	-0.24983	4.56863	0.332051
O	0.186171	5.492567	1.104681
C	1.545528	2.905446	-0.31111
S	2.001826	1.063978	-0.26054
O	-1.09931	4.800576	-0.62399
H	0.498225	2.987512	1.568172

$^{5}A'_{3}$

Fe	-0.17969	0.284446	0.810977
N	0.064653	-0.4113	2.817569
C	1.129769	-0.02831	3.533464
N	1.111168	-0.65484	4.745113
C	-0.01212	-1.48588	4.816993
C	-0.65017	-1.32001	3.609338
H	1.88836	0.654356	3.182113
H	1.803335	-0.53868	5.469402
N	-2.36308	-0.17656	0.763592
C	-3.0215	-0.57959	-0.32699
N	-4.35947	-0.66695	-0.05486
C	-3.32599	0.000807	1.767142
C	-4.57737	-0.30154	1.276727
C	-5.9292	-0.29046	1.909536
H	-6.61768	0.390013	1.3934
H	-5.85342	0.042066	2.947817
H	-3.05151	0.320959	2.757859
H	-2.58382	-0.78086	-1.28947
H	-5.07227	-0.94156	-0.71353
H	-6.38764	-1.28712	1.913923
N	0.30093	-1.39872	-0.42385
C	1.334563	-1.36516	-1.27303
N	1.46134	-2.57755	-1.88494
C	0.469088	-3.43998	-1.40665
C	-0.24383	-2.68775	-0.50151
C	0.331468	-4.855	-1.86088
H	1.229164	-5.44447	-1.63717
H	0.149374	-4.92347	-2.9406
H	-1.0886	-2.98044	0.097502
H	1.977003	-0.51463	-1.42752
H	2.170142	-2.81158	-2.56351
H	-0.51049	-5.33193	-1.35267
N	-0.82497	2.348901	0.366229
C	0.110616	3.414808	0.833358
C	-0.36806	4.818699	0.43557
O	0.076796	5.835064	1.07372
C	1.533585	3.163421	0.287516
S	2.082896	1.371679	0.552327
O	-1.19431	4.934696	-0.56405
H	0.151651	3.37259	1.926149

H	1.411544	3.212119	-1.35195	H	1.576947	3.387763	-0.78359
H	2.368376	3.476621	0.122159	H	2.230812	3.819858	0.809614
H	-1.60567	2.227902	0.699973	H	-1.75881	2.477629	0.755378
H	-1.03233	2.235931	-0.85907	H	-0.90513	2.353169	-0.6582
C	7.172075	7.777781	2.242785	C	7.119178	8.123772	2.122637
C	5.858167	7.03245	2.377235	C	5.759037	7.487277	2.334166
C	4.798457	7.241257	1.481305	C	4.772484	7.507123	1.336644
C	5.662579	6.090778	3.406795	C	5.446704	6.837583	3.54491
C	3.588423	6.543687	1.598289	C	3.522095	6.902187	1.52726
C	4.464144	5.389276	3.537423	C	4.208118	6.231319	3.75024
C	3.417083	5.611549	2.631729	C	3.235846	6.2582	2.739661
O	2.248792	4.880557	2.803252	O	2.028991	5.625207	2.996828
H	7.126678	8.521265	1.440426	H	7.168691	8.645422	1.161292
H	8.000186	7.094803	2.013404	H	7.920027	7.373089	2.132151
H	4.920761	7.954536	0.670435	H	4.985098	7.993651	0.388227
H	6.463504	5.905596	4.117727	H	6.189021	6.80609	4.338177
H	2.791145	6.705142	0.877933	H	2.783676	6.908388	0.729895
H	4.318479	4.668731	4.334326	H	3.972153	5.732157	4.683169
H	1.510647	5.178099	2.209162	H	1.347356	5.762079	2.289394
C	-0.11591	9.153026	1.002653	C	-0.04513	9.423618	0.244501
N	-0.91646	7.992108	0.616873	N	-0.90916	8.2513	0.11394
C	-2.13258	8.079119	0.073294	C	-2.13027	8.289437	-0.42308
N	-2.77017	9.276311	0.003759	N	-2.71789	9.479792	-0.711
N	-2.72208	6.975292	-0.41686	N	-2.77376	7.140421	-0.69281
H	0.877499	8.797323	1.279187	H	0.936545	9.080639	0.57408
H	-0.53533	9.674592	1.872705	H	-0.41709	10.13115	0.996826
H	-0.5064	7.04923	0.783382	H	-0.54327	7.341198	0.464988
H	-2.40846	10.08907	0.473931	H	-2.32108	10.3486	-0.39413
H	-3.61448	9.387121	-0.53285	H	-3.56118	9.525934	-1.2585
H	-2.16314	6.103205	-0.5168	H	-2.24482	6.245002	-0.64701
H	-3.6957	6.974863	-0.67478	H	-3.75599	7.133444	-0.91556
C	-1.28608	0.683296	-5.74693	C	-0.85266	0.286747	-5.18762
S	-2.20695	0.682692	-4.08359	S	-1.91646	0.507148	-3.63004
H	-1.98174	0.936719	-6.5472	H	-1.47558	0.41112	-6.07367
H	-0.91259	-0.33314	-5.8817	H	-0.46706	-0.73302	-5.14122
C	0.43417	4.781652	-5.8678	C	0.797012	4.080146	-5.86718
C	1.416176	3.742584	-5.42977	C	1.739696	3.202192	-5.1083
N	1.231064	2.945611	-4.29798	N	1.492873	2.774087	-3.80327
C	2.633785	3.346254	-5.94903	C	2.97426	2.669884	-5.43102
C	2.332398	2.120001	-4.18357	C	2.576312	2.021469	-3.39743
N	3.201045	2.336359	-5.16891	N	3.491661	1.936076	-4.36184
H	-0.55542	4.358761	-6.08592	H	-0.20106	3.634658	-5.97238
H	0.29326	5.563302	-5.1088	H	0.666678	5.057413	-5.38314
H	3.13601	3.72292	-6.82558	H	3.522287	2.774555	-6.35358
H	2.451388	1.420134	-3.37198	H	2.658702	1.605221	-2.40502

H	0.421144	2.963197	-3.67854
C	-5.9898	4.159641	-5.50406
C	-4.68484	3.917228	-4.79968
C	-4.03179	4.963464	-4.13013
C	-4.09997	2.644	-4.77268
C	-2.85021	4.734659	-3.43278
C	-2.90799	2.393655	-4.08126
C	-2.2939	3.446243	-3.38657
O	-1.12867	3.235412	-2.65859
H	-5.9851	5.111603	-6.04718
H	-6.82968	4.197384	-4.79656
H	-4.45127	5.965132	-4.16551
H	-4.58166	1.822774	-5.29368
H	-2.33525	5.549174	-2.93522
H	-1.03561	3.940378	-1.92958
H	7.434417	8.303039	3.169656
H	-0.45427	1.385177	-5.70561
H	-6.21208	3.368273	-6.22694
H	-0.00746	9.859273	0.171763
H	0.792829	5.2693	-6.77901
H	-1.80145	-0.86205	3.254177
C	-0.67282	-0.65795	6.086863
H	-1.65688	-1.13285	6.053931
H	0.02197	-1.36944	6.550381
H	-0.74758	0.212853	6.750155

$^{5}C_{3}$

Fe	-0.28675	0.026809	0.231596
N	-0.05419	0.290691	2.433321
C	0.810335	1.064249	3.100524
N	0.664989	0.869087	4.446178
C	-0.33485	-0.08042	4.664237
C	-0.76828	-0.42714	3.403306
H	1.527335	1.752609	2.686499
H	1.194143	1.34626	5.160284
N	-2.38973	-0.40675	0.002533
C	-2.81074	-0.6451	-1.24523
N	-4.1486	-0.9156	-1.2304
C	-3.50754	-0.53054	0.837183
C	-4.61648	-0.84897	0.086484
C	-6.04022	-1.09573	0.460241
H	-6.72259	-0.39589	-0.03811
H	-6.17121	-0.97259	1.53838
H	-3.44067	-0.37801	1.900639
H	-2.19325	-0.59502	-2.12839

H	0.637259	2.942552	-3.27346
C	-5.65106	3.626307	-5.81669
C	-4.4164	3.521644	-4.96279
C	-3.86548	4.666014	-4.36254
C	-3.78993	2.289262	-4.73604
C	-2.73798	4.575099	-3.55294
C	-2.65276	2.176801	-3.92448
C	-2.13323	3.329353	-3.31651
O	-1.02097	3.259653	-2.48651
H	-5.49978	4.307034	-6.66382
H	-6.50588	4.010967	-5.24562
H	-4.32342	5.635361	-4.53923
H	-4.19668	1.390622	-5.18861
H	-2.3052	5.463498	-3.10539
H	-1.03625	4.009711	-1.79321
H	7.350867	8.852425	2.909668
H	-0.02985	1.000213	-5.1756
H	-5.94054	2.653569	-6.22593
H	0.075295	9.940888	-0.71395
H	1.185907	4.26166	-6.87343
H	-1.55319	-1.78704	3.256589
C	-0.33028	-2.32406	6.0102
H	-1.24955	-2.88928	5.836938
H	0.466242	-3.04684	6.22582
H	-0.48003	-1.71515	6.91043

$^{3}D_{3}$

Fe	-0.17207	0.091719	0.084752
N	-0.12945	0.154733	2.215967
C	0.898323	-0.25413	2.96536
N	0.658223	0.034355	4.278418
C	-0.58328	0.66378	4.387172
C	-1.05883	0.72599	3.096047
H	1.791076	-0.7291	2.600745
H	1.292295	-0.14701	5.041752
N	-2.20249	-0.73461	0.131567
C	-2.97593	-1.27831	1.074286
N	-4.10292	-1.80799	0.500833
C	-2.85657	-0.92687	-1.08981
C	-4.04377	-1.59284	-0.88003
C	-5.10872	-2.04478	-1.82289
H	-5.23364	-3.1349	-1.81141
H	-4.84324	-1.7535	-2.84197
H	-2.41002	-0.58135	-2.00713
H	-2.76565	-1.31229	2.128898

H	-4.70738	-1.11318	-2.04602	H	-4.84778	-2.28069	0.988952
H	-6.36085	-2.11294	0.20287	H	-6.08247	-1.59469	-1.59162
N	0.70185	-1.89835	0.341482	N	0.706911	-1.74367	0.098185
C	1.043521	-2.4988	-0.80463	C	1.650572	-2.06089	-0.79169
N	1.716687	-3.65311	-0.5288	N	1.95364	-3.38549	-0.66908
C	1.81622	-3.80611	0.85916	C	1.162325	-3.94965	0.337304
C	1.178693	-2.70443	1.381315	C	0.391512	-2.90915	0.803394
C	2.499784	-4.96582	1.503599	C	1.232677	-5.391	0.718481
H	3.556614	-5.02923	1.216055	H	2.231383	-5.67404	1.073785
H	2.025273	-5.91971	1.24127	H	0.980868	-6.04844	-0.1229
H	1.034708	-2.43704	2.41338	H	-0.36558	-2.92113	1.56712
H	0.828375	-2.09731	-1.7807	H	2.109451	-1.3716	-1.48278
H	2.098935	-4.28295	-1.21718	H	2.636313	-3.876	-1.22596
H	2.460325	-4.86599	2.591324	H	0.524023	-5.60124	1.523788
N	-0.71799	2.236146	-0.04141	N	-0.8729	2.070503	0.044085
C	0.409661	3.167033	0.210613	C	0.132687	3.039068	0.554306
C	-0.0367	4.630965	0.099611	C	-0.36031	4.487908	0.487446
O	0.299568	5.444838	1.037141	O	0.126507	5.319997	1.334744
C	1.584355	2.907749	-0.74611	C	1.456696	2.89583	-0.20703
S	2.192992	1.113949	-0.72231	S	2.178717	1.172886	0.188779
O	-0.74784	4.976088	-0.92067	O	-1.2361	4.801483	-0.40485
H	0.747917	3.019118	1.234704	H	0.308478	2.822948	1.612397
H	1.335407	3.114308	-1.78938	H	1.339538	2.939566	-1.29175
H	2.442184	3.521244	-0.45804	H	2.18783	3.627726	0.13657
H	-1.52525	2.438947	0.545618	H	-1.76385	2.151147	0.5306
H	-0.98619	2.21745	-1.03166	H	-1.0272	2.167937	-0.96689
C	7.1793	7.384722	2.580172	C	7.314495	7.334733	2.096688
C	5.880858	6.600976	2.575715	C	5.987899	6.616241	2.257635
C	4.803621	6.977934	1.757794	C	4.864979	6.964187	1.490883
C	5.704716	5.476526	3.404626	C	5.844615	5.557496	3.176074
C	3.592949	6.274765	1.767904	C	3.644705	6.287723	1.62596
C	4.502571	4.765517	3.428025	C	4.63624	4.875518	3.323528
C	3.438139	5.165208	2.611177	C	3.527051	5.238357	2.547763
O	2.251587	4.430458	2.663063	O	2.349212	4.515907	2.723655
H	7.279245	7.994063	1.676195	H	7.21017	8.225157	1.468645
H	8.048686	6.719866	2.633543	H	8.066733	6.687364	1.627447
H	4.913163	7.831682	1.094522	H	4.943967	7.770915	0.767331
H	6.5222	5.153022	4.04302	H	6.694706	5.264002	3.786103
H	2.78087	6.57207	1.111188	H	2.797329	6.560281	1.002419
H	4.375823	3.903006	4.07297	H	4.531962	4.063966	4.034835
H	1.516303	4.87861	2.161667	H	1.564791	4.923129	2.271532
C	-0.07018	8.997696	1.402089	C	-0.04018	8.750268	1.426322
N	-0.82418	7.924416	0.764097	N	-1.00296	7.741199	1.011458
C	-1.96728	8.102256	0.100275	C	-2.19527	7.992766	0.476266
N	-2.60365	9.301137	0.127664	N	-2.69843	9.253305	0.460962

N	-2.48311	7.081494	-0.6059	N	-2.89888	6.977552	-0.05947
H	0.887603	8.587165	1.724433	H	0.896937	8.241303	1.655287
H	-0.57865	9.392133	2.291664	H	-0.35827	9.283249	2.331941
H	-0.42068	6.965871	0.836415	H	-0.66974	6.759719	1.115014
H	-2.30551	10.03689	0.746062	H	-2.24858	10.00005	0.964027
H	-3.37394	9.496685	-0.49018	H	-3.51574	9.483163	-0.0799
H	-1.8958	6.243802	-0.77405	H	-2.40978	6.082132	-0.22291
H	-3.41889	7.115749	-0.97643	H	-3.86634	7.081985	-0.31746
C	-2.65241	0.485044	-6.25183	C	-2.52672	0.60571	-6.13088
S	-2.05028	0.964606	-4.52454	S	-1.94515	1.082037	-4.39628
H	-3.70102	0.184667	-6.24523	H	-3.53045	0.17917	-6.1135
H	-2.02981	-0.36151	-6.54516	H	-1.81482	-0.14741	-6.47178
C	0.375869	4.918915	-5.65335	C	0.057933	4.813781	-5.72279
C	1.445387	3.885459	-5.50778	C	1.303939	4.17599	-5.19786
N	1.327424	2.778269	-4.66831	N	1.298911	3.15414	-4.2498
C	2.690028	3.756677	-6.09393	C	2.639574	4.392945	-5.47826
C	2.48354	2.035644	-4.78744	C	2.610679	2.799077	-4.00244
N	3.333873	2.602604	-5.64141	N	3.449122	3.536175	-4.73041
H	-0.57606	4.486875	-5.98685	H	-0.6178	4.081238	-6.18314
H	0.178684	5.448531	-4.71145	H	-0.51163	5.32831	-4.9379
H	3.159108	4.413986	-6.80853	H	3.06594	5.102931	-6.1689
H	2.638659	1.119681	-4.24394	H	2.887725	2.026612	-3.30223
H	0.517809	2.551558	-4.10112	H	0.468198	2.760622	-3.82305
C	-6.06218	4.444983	-5.3167	C	-6.3278	4.140928	-4.97631
C	-4.70376	4.165628	-4.7477	C	-4.94234	3.977046	-4.42592
C	-4.01353	5.15442	-4.03633	C	-4.37071	4.979374	-3.63347
C	-4.11328	2.901444	-4.89275	C	-4.2118	2.802598	-4.66387
C	-2.78851	4.867531	-3.44054	C	-3.12025	4.788488	-3.05132
C	-2.87861	2.600033	-4.31339	C	-2.95153	2.598334	-4.09716
C	-2.23766	3.584488	-3.54479	C	-2.42857	3.5893	-3.2524
O	-1.04183	3.281868	-2.90799	O	-1.20454	3.374002	-2.62696
H	-6.17305	5.497397	-5.60032	H	-6.57137	5.194791	-5.1499
H	-6.8585	4.221896	-4.59239	H	-7.08597	3.744582	-4.2861
H	-4.43502	6.152945	-3.96075	H	-4.90703	5.911008	-3.4774
H	-4.63596	2.146385	-5.4698	H	-4.6481	2.038411	-5.29758
H	-2.22942	5.638646	-2.92457	H	-2.65938	5.570507	-2.45954
H	-0.82188	3.995976	-2.23204	H	-1.08847	4.040672	-1.88846
H	7.236705	8.063145	3.442174	H	7.719642	7.652857	3.064843
H	-2.49135	1.31554	-6.93972	H	-2.48656	1.475685	-6.78741
H	-6.26352	3.841267	-6.20834	H	-6.45483	3.612412	-5.9275
H	0.128954	9.820251	0.705445	H	0.15307	9.479178	0.630381
H	0.680086	5.664427	-6.39435	H	0.314556	5.553721	-6.48703
O	-0.08962	-0.05767	-1.77152	H	-1.99321	1.130941	2.748061
O	1.277611	0.451549	-2.1755	C	-1.15042	1.131834	5.68566
H	-1.52314	-1.14292	3.126746	H	-2.13534	1.577622	5.526136

C	-0.74859	-0.53443	6.02456
H	-1.53619	-1.28796	5.944764
H	0.085408	-0.98464	6.576827
H	-1.14074	0.292005	6.630133

$^{5}E_{3}$

Fe	-0.15256	0.088403	0.255167
N	-0.18095	0.053459	2.43061
C	0.805011	0.541144	3.190507
N	0.512214	0.324925	4.507248
C	-0.71707	-0.33493	4.605061
C	-1.13218	-0.49454	3.302547
H	1.691666	1.021149	2.809361
H	1.093592	0.603742	5.282421
N	-2.17226	-0.63463	-0.1074
C	-2.40483	-1.6363	-0.95746
N	-3.75012	-1.78441	-1.14359
C	-3.42392	-0.12555	0.258892
C	-4.42162	-0.82817	-0.37842
C	-5.90843	-0.70428	-0.3493
H	-6.31692	-0.47414	-1.34096
H	-6.20365	0.104041	0.324525
H	-3.52259	0.691383	0.953157
H	-1.65197	-2.23985	-1.43143
H	-4.18574	-2.4461	-1.76713
H	-6.39017	-1.62375	0.005641
N	0.684292	-1.94767	0.016611
C	1.532387	-2.23688	-0.97262
N	2.060594	-3.48479	-0.77746
C	1.529999	-4.02177	0.399324
C	0.675222	-3.05181	0.87481
C	1.903898	-5.36916	0.921677
H	2.976558	-5.43931	1.141312
H	1.65671	-6.16936	0.212693
H	0.077618	-3.06194	1.770178
H	1.770512	-1.57352	-1.78605
H	2.740061	-3.93213	-1.37305
H	1.364398	-5.57302	1.85023
N	-0.90719	2.29679	0.091429
C	0.177151	3.26532	0.412294
C	-0.34828	4.710289	0.437236
O	0.065283	5.522699	1.336764
C	1.338384	3.246743	-0.59473
S	2.174224	1.528781	-0.76179
O	-1.18926	5.050554	-0.48997

H	-1.26964	0.309366	6.401362
H	-0.51484	1.893424	6.153457
O	3.051039	0.723747	-1.17516
O	-0.10328	0.144503	-1.56342

$^{5}B_{3}$

Fe	-0.25301	-0.09173	0.305053
N	-0.16199	0.067257	2.518639
C	0.945692	0.316001	3.222856
N	0.633698	0.43564	4.54903
C	-0.74095	0.256373	4.713219
C	-1.21757	0.027429	3.440808
H	1.932741	0.438131	2.81214
H	1.290501	0.654212	5.282803
N	-2.20741	-0.79384	-0.2077
C	-2.99232	-1.65275	0.449019
N	-4.10628	-1.91414	-0.29745
C	-2.8412	-0.50306	-1.42125
C	-4.0292	-1.19375	-1.49467
C	-5.0736	-1.25272	-2.55818
H	-5.20412	-2.26985	-2.9478
H	-4.77692	-0.61235	-3.39216
H	-2.38558	0.139365	-2.15637
H	-2.78568	-2.1003	1.405491
H	-4.85538	-2.53724	-0.03738
H	-6.04904	-0.90382	-2.19702
N	0.664224	-2.07356	0.328939
C	1.38206	-2.54905	-0.69196
N	1.899947	-3.76943	-0.36129
C	1.497609	-4.09816	0.937108
C	0.73062	-3.03113	1.347319
C	1.894419	-5.36644	1.616709
H	2.983407	-5.45319	1.716948
H	1.538576	-6.25154	1.07483
H	0.24316	-2.87829	2.294675
H	1.544661	-2.04344	-1.62772
H	2.490959	-4.33299	-0.95308
H	1.467699	-5.40167	2.62238
N	-1.00903	2.116732	0.384928
C	0.123019	3.039799	0.665324
C	-0.30306	4.501058	0.498765
O	0.230252	5.381765	1.266485
C	1.327514	2.733904	-0.24825
S	2.051084	1.023764	0.068761
O	-1.18082	4.784773	-0.40682

H	0.578173	3.02179	1.39855
H	1.043254	3.500971	-1.61195
H	2.13233	3.914538	-0.25051
H	-1.70918	2.42023	0.703882
H	-1.1791	2.335088	-0.894
C	7.194261	7.559613	2.463757
C	5.869312	6.823432	2.541389
C	4.777841	7.179778	1.734492
C	5.689848	5.751196	3.438284
C	3.551002	6.504954	1.816032
C	4.475745	5.071549	3.532475
C	3.393689	5.44687	2.722853
O	2.210822	4.732972	2.860304
H	7.191659	8.29772	1.655272
H	8.02724	6.869577	2.281783
H	4.885522	7.996044	1.024965
H	6.516484	5.446059	4.074341
H	2.726886	6.788333	1.166447
H	4.343978	4.249727	4.227019
H	1.44804	5.118283	2.355269
C	-0.09223	9.011492	1.304632
N	-1.0305	7.979066	0.885637
C	-2.1985	8.213138	0.287647
N	-2.70293	9.472335	0.226008
N	-2.86881	7.188748	-0.26864
H	0.839472	8.519839	1.587511
H	-0.4489	9.570144	2.179873
H	-0.71186	7.001475	1.045911
H	-2.29395	10.22471	0.754832
H	-3.49268	9.690033	-0.35896
H	-2.36478	6.292214	-0.39063
H	-3.82655	7.282229	-0.56456
C	-1.73824	0.545071	-5.4958
S	-2.27058	0.861763	-3.70005
H	-2.5883	0.68173	-6.165
H	-1.39928	-0.4917	-5.5268
C	0.396182	4.841333	-5.69436
C	1.150972	3.564298	-5.50504
N	0.929541	2.698309	-4.43343
C	2.146448	2.960783	-6.25069
C	1.78428	1.624165	-4.57892
N	2.537641	1.752049	-5.66991
H	-0.68348	4.675127	-5.80054
H	0.53811	5.533673	-4.85333
H	2.602831	3.316687	-7.16035

H	0.428348	2.902671	1.705075
H	1.035273	2.763147	-1.30471
H	2.125158	3.461245	-0.08196
H	-1.72913	2.166998	1.101969
H	-1.41334	2.312124	-0.53343
C	7.389001	7.299479	1.820383
C	6.05318	6.623941	2.068748
C	4.944397	6.859518	1.241041
C	5.880118	5.735006	3.147969
C	3.70786	6.241914	1.474193
C	4.655806	5.112883	3.393956
C	3.558695	5.363637	2.557475
O	2.365194	4.712718	2.844051
H	7.391044	7.835005	0.86563
H	8.208599	6.571225	1.795178
H	5.047687	7.528931	0.391276
H	6.720097	5.527449	3.805693
H	2.871716	6.423031	0.804371
H	4.529311	4.431854	4.228135
H	1.605275	5.026401	2.286413
C	0.06061	8.900401	1.294729
N	-0.86446	7.827473	0.957774
C	-2.11226	8.008914	0.532264
N	-2.68289	9.241099	0.553754
N	-2.80766	6.952483	0.070922
H	1.043557	8.455186	1.455075
H	-0.22276	9.419826	2.219618
H	-0.48656	6.857435	1.049776
H	-2.23036	10.01748	1.00683
H	-3.55602	9.419927	0.085968
H	-2.29414	6.075059	-0.1312
H	-3.79983	7.00646	-0.0912
C	-2.6801	1.145924	-6.56156
S	-2.05824	1.324013	-4.78541
H	-3.67642	0.703776	-6.59802
H	-1.96605	0.478259	-7.04608
C	-0.15231	4.693036	-5.69579
C	1.148793	4.099147	-5.26553
N	1.241505	3.114994	-4.28595
C	2.451736	4.324174	-5.66941
C	2.572174	2.786905	-4.14296
N	3.336184	3.504589	-4.96514
H	-0.85673	3.926616	-6.03996
H	-0.64561	5.245505	-4.8864
H	2.806464	5.014729	-6.41764

H	1.795816	0.806431	-3.87927
H	0.237349	2.821072	-3.69665
C	-6.02921	4.24479	-5.31386
C	-4.70792	4.061318	-4.64467
C	-4.03469	5.156093	-4.08502
C	-4.14483	2.786238	-4.53014
C	-2.83753	4.968626	-3.40553
C	-2.94029	2.575753	-3.85563
C	-2.29942	3.679898	-3.27655
O	-1.11919	3.517216	-2.56407
H	-6.07737	5.187348	-5.87121
H	-6.85361	4.263061	-4.58667
H	-4.4513	6.153671	-4.19073
H	-4.66255	1.935059	-4.96077
H	-2.29668	5.809596	-2.9844
H	-1.07182	4.211422	-1.82372
H	7.414034	8.092172	3.398166
H	-0.91748	1.21171	-5.75599
H	-6.24463	3.43573	-6.01953
H	0.123635	9.71546	0.492546
H	0.745129	5.347074	-6.59948
H	-2.032	-0.95739	2.934198
C	-1.33207	-0.72608	5.907739
H	-2.28662	-1.22995	5.735111
H	-0.69191	-1.41544	6.472398
H	-1.52761	0.143949	6.547037
O	0.864408	0.642767	-1.50525
O	2.019099	0.937232	0.821645

$^3A_3$

Fe	-0.05023	-0.05481	-0.12274
N	-0.08005	0.05675	2.209773
C	1.07614	0.203152	2.864307
N	0.85349	0.198911	4.215973
C	-0.51301	0.037467	4.44934
C	-1.07432	-0.05105	3.192994
H	2.044556	0.285632	2.399265
H	1.564481	0.309909	4.922538
N	-2.08973	-0.0602	-0.48767
C	-2.59686	-0.64045	-1.57669
N	-3.9533	-0.47131	-1.59698
C	-3.16635	0.497599	0.211542
C	-4.34038	0.249049	-0.46433
C	-5.75969	0.601444	-0.16643
H	-6.20617	1.21011	-0.96246

H	2.920974	2.040238	-3.44893
H	0.456977	2.716491	-3.78424
C	-6.53065	4.233937	-4.75116
C	-5.12529	4.071753	-4.27036
C	-4.56311	4.985472	-3.37265
C	-4.36141	2.974746	-4.68739
C	-3.28561	4.772222	-2.86565
C	-3.07333	2.751287	-4.20039
C	-2.55182	3.641217	-3.24512
O	-1.30539	3.402409	-2.69139
H	-6.82609	5.288205	-4.79671
H	-7.24804	3.730579	-4.08709
H	-5.12853	5.863939	-3.07494
H	-4.79327	2.286514	-5.40553
H	-2.8298	5.488224	-2.1942
H	-1.14175	4.012545	-1.91473
H	7.62662	8.02652	2.608316
H	-2.67022	2.118176	-7.05589
H	-6.67255	3.81093	-5.75177
H	0.146083	9.631658	0.482376
H	0.013611	5.391543	-6.52191
O	0.400566	-0.01695	-1.7066
O	-0.10282	0.905251	-2.52371
H	-2.23321	-0.15031	3.129617
C	-1.4222	0.32982	6.039265
H	-2.49603	0.165081	5.919553
H	-1.0461	-0.4298	6.735564
H	-1.2885	1.310142	6.512879

$^5A_3$

Fe	-0.03056	-0.10329	-0.0345
N	-0.03255	-0.07538	2.174477
C	1.096878	0.120492	2.862182
N	0.840289	0.025617	4.201577
C	-0.5171	-0.24947	4.388107
C	-1.04145	-0.30942	3.115958
H	2.062737	0.303313	2.418729
H	1.524168	0.143227	4.9333
N	-2.23021	-0.16287	-0.32898
C	-2.75914	-0.82666	-1.35896
N	-4.11454	-0.6383	-1.38726
C	-3.29446	0.47107	0.323627
C	-4.47894	0.187286	-0.32051
C	-5.88854	0.59679	-0.04997
H	-6.32109	1.154375	-0.88993

H	-5.81661	1.177556	0.760656	H	-5.92828	1.243339	0.830362
H	-3.02855	1.021567	1.141744	H	-3.13894	1.076693	1.200315
H	-2.01877	-1.13465	-2.33649	H	-2.20444	-1.40586	-2.07559
H	-4.5699	-0.79844	-2.32453	H	-4.74351	-1.01574	-2.07922
H	-6.38494	-0.29107	-0.03947	H	-6.53632	-0.26698	0.144666
N	0.046443	-2.07063	-0.1399	N	0.165119	-2.30647	0.014084
C	0.344659	-2.85112	-1.18368	C	1.287673	-2.92968	-0.34899
N	0.402728	-4.15451	-0.7751	N	1.14072	-4.28067	-0.18743
C	0.132273	-4.21654	0.594197	C	-0.14091	-4.54018	0.30724
C	-0.08937	-2.91178	0.970274	C	-0.72928	-3.30063	0.423928
C	0.127159	-5.49254	1.368465	C	-0.63658	-5.9165	0.60419
H	1.104909	-5.9897	1.343077	H	-0.02375	-6.41787	1.36374
H	-0.61777	-6.20266	0.987848	H	-0.64391	-6.55286	-0.28969
H	-0.32101	-2.52011	1.943701	H	-1.71675	-3.05617	0.775374
H	0.5033	-2.4988	-2.18783	H	2.184277	-2.45179	-0.70614
H	0.63067	-4.93933	-1.36491	H	1.844994	-4.9737	-0.38835
H	-0.11271	-5.29271	2.415945	H	-1.66023	-5.87139	0.985061
N	-0.07351	2.019358	-0.07592	N	-0.03434	2.18816	-0.0419
C	1.228513	2.651385	0.299483	C	1.279486	2.790358	0.301552
C	1.181758	4.157705	0.063333	C	1.271812	4.29343	0.024767
O	1.809845	4.926195	0.87526	O	1.903834	5.077863	0.818291
C	2.401259	2.031227	-0.47862	C	2.453253	2.141191	-0.46814
S	2.313207	0.135654	-0.41418	S	2.483115	0.250043	-0.33293
O	0.525442	4.600702	-0.96659	O	0.633415	4.725761	-1.02342
H	1.384892	2.474102	1.364039	H	1.446406	2.642482	1.370577
H	2.399661	2.358757	-1.52231	H	2.41911	2.422383	-1.52477
H	3.341226	2.346857	-0.02151	H	3.388207	2.519638	-0.04908
H	-0.80549	2.289597	0.579856	H	-0.76631	2.487937	0.598497
H	-0.35106	2.297782	-1.02029	H	-0.30723	2.398404	-1.00326
C	9.117206	4.813437	2.279586	C	9.208152	4.953644	2.138239
C	7.625976	4.551548	2.374431	C	7.714963	4.72116	2.270852
C	6.725761	5.076477	1.434357	C	6.807592	5.205896	1.316253
C	7.094781	3.753937	3.407154	C	7.188924	3.990862	3.354725
C	5.349818	4.820181	1.510898	C	5.429647	4.974513	1.427218
C	5.727758	3.490974	3.498236	C	5.820166	3.753067	3.480373
C	4.844835	4.023399	2.548378	C	4.929452	4.243725	2.514911
O	3.493738	3.724639	2.682273	O	3.578463	3.973108	2.686885
H	9.340741	5.564231	1.514797	H	9.429281	5.670613	1.340858
H	9.669357	3.901525	2.017177	H	9.740496	4.023171	1.900659
H	7.104108	5.6901	0.621086	H	7.181752	5.767573	0.464346
H	7.76455	3.333288	4.15247	H	7.864557	3.603271	4.112661
H	4.676346	5.218382	0.756893	H	4.750058	5.340035	0.662352
H	5.325508	2.879911	4.298391	H	5.421377	3.193463	4.318974
H	2.917263	4.235197	2.055502	H	2.999232	4.437041	2.026961
C	2.62277	8.343944	0.900537	C	2.73166	8.509643	0.74032

N	1.516321	7.54897	0.384246	N	1.627477	7.695158	0.249613
C	0.453883	8.043217	-0.2462	C	0.563083	8.171152	-0.39216
N	0.246933	9.383377	-0.31662	N	0.354049	9.509306	-0.49525
N	-0.41778	7.195497	-0.82618	N	-0.30697	7.308688	-0.95108
H	3.405459	7.65617	1.22369	H	3.518592	7.83506	1.080645
H	2.331225	8.947738	1.769933	H	2.439592	9.136345	1.593229
H	1.587739	6.516886	0.537489	H	1.699552	6.667373	0.4319
H	0.815561	10.02644	0.208796	H	0.92081	10.16595	0.015046
H	-0.46145	9.772346	-0.91662	H	-0.35499	9.881872	-1.10472
H	-0.15719	6.196089	-0.91398	H	-0.0427	6.307086	-1.01447
H	-1.32087	7.507961	-1.14328	H	-1.21125	7.611438	-1.27414
C	-0.55631	1.019557	-6.52234	C	-0.61855	0.866645	-6.14857
S	-1.50805	1.070656	-4.87647	S	-1.67572	1.092275	-4.58193
H	-1.12682	1.553902	-7.28186	H	-1.12843	1.337292	-6.98884
H	-0.47735	-0.03674	-6.78343	H	-0.5468	-0.21058	-6.30593
C	2.04701	4.137172	-6.06983	C	2.037691	4.130078	-6.11186
C	2.792784	2.915273	-5.64418	C	2.786853	2.936113	-5.61703
N	2.441291	2.172269	-4.51551	N	2.433539	2.253111	-4.45133
C	3.909496	2.282719	-6.1551	C	3.908788	2.282481	-6.08784
C	3.353514	1.14133	-4.39375	C	3.35281	1.236626	-4.26764
N	4.254513	1.179782	-5.37183	N	4.257004	1.226402	-5.24358
H	0.990892	3.93464	-6.28984	H	0.982271	3.910922	-6.3202
H	2.072229	4.922521	-5.30229	H	2.060029	4.957681	-5.38999
H	4.486065	2.551339	-7.02544	H	4.487301	2.505716	-6.96964
H	3.329649	0.424681	-3.58869	H	3.336883	0.574106	-3.41621
H	1.640533	2.370821	-3.91921	H	1.632683	2.480372	-3.86455
C	-4.27467	5.473757	-5.87891	C	-4.26867	5.53184	-5.88549
C	-3.07928	4.862804	-5.20704	C	-3.08543	4.916297	-5.19233
C	-2.21567	5.637321	-4.41788	C	-2.16286	5.709257	-4.49264
C	-2.81459	3.493583	-5.32512	C	-2.88749	3.529821	-5.20342
C	-1.15789	5.049984	-3.73006	C	-1.10846	5.131046	-3.79087
C	-1.75039	2.886459	-4.64963	C	-1.82998	2.931621	-4.50917
C	-0.93755	3.665475	-3.81145	C	-0.95088	3.735726	-3.76662
O	0.083222	3.08766	-3.06048	O	0.071464	3.169378	-3.01383
H	-4.06341	6.486171	-6.24111	H	-4.02147	6.50751	-6.31836
H	-5.12721	5.550281	-5.1899	H	-5.10419	5.690288	-5.18969
H	-2.37505	6.709626	-4.34562	H	-2.27133	6.790263	-4.50437
H	-3.45574	2.876336	-5.94606	H	-3.57403	2.894877	-5.75396
H	-0.48084	5.655554	-3.13935	H	-0.38602	5.750353	-3.27193
H	0.359746	3.708542	-2.30591	H	0.381528	3.811707	-2.28077
H	9.524039	5.174612	3.231947	H	9.639366	5.344003	3.068089
H	0.435588	1.449563	-6.39078	H	0.371645	1.29317	-5.99412
H	-4.60574	4.878403	-6.73578	H	-4.63973	4.894867	-6.69477
H	3.043591	9.000975	0.130257	H	3.147069	9.146371	-0.04965
H	2.498251	4.550887	-6.97652	H	2.487937	4.492714	-7.04057

O	0.24786	-0.41294	-2.67675
H	1.161477	-0.22407	-2.3387
H	-0.05765	0.268424	-3.31744
H	-2.10848	-0.20357	2.935233
C	-1.10966	-0.01373	5.816834
H	-2.19001	-0.16575	5.74884
H	-0.69698	-0.83675	6.413432
H	-0.94029	0.916545	6.373465

$^{3}R_{2}$

Fe	-0.11826	-0.36075	0.183164
N	-0.23776	-0.46765	2.275095
C	0.714034	-0.90465	3.112404
N	0.26596	-0.82495	4.396975
C	-1.03292	-0.30997	4.401786
C	-1.33064	-0.09542	3.073482
H	1.686121	-1.27203	2.83597
H	0.7884	-1.09394	5.219039
N	-2.09433	-0.37833	-0.03865
C	-2.88552	-1.45547	-0.12006
N	-4.17728	-1.05357	-0.29059
C	-2.92458	0.746985	-0.16319
C	-4.23182	0.341341	-0.3224
C	-5.50019	1.106726	-0.50175
H	-5.97892	0.879896	-1.46161
H	-5.30084	2.180475	-0.47838
H	-2.53164	1.747002	-0.12043
H	-2.56656	-2.48047	-0.06072
H	-4.97291	-1.66906	-0.383
H	-6.22358	0.8864	0.291875
N	0.183391	-2.46605	-0.28575
C	0.531435	-2.97931	-1.47293
N	0.620073	-4.33899	-1.38018
C	0.315177	-4.73074	-0.07561
C	0.045449	-3.55366	0.590771
C	0.321849	-6.15417	0.372889
H	1.314007	-6.60885	0.268298
H	-0.38616	-6.76506	-0.19931
H	-0.23068	-3.41647	1.621925
H	0.703702	-2.42396	-2.37775
H	0.866117	-4.96488	-2.13412
H	0.038479	-6.21949	1.425897
O	0.044973	0.266403	-1.81394
H	0.89525	0.478721	-2.23704

O	0.101317	-0.15799	-2.21749
H	1.087975	-0.11809	-2.24926
H	-0.33437	0.394187	-2.91054
H	-2.05479	-0.51556	2.81748
C	-1.13716	-0.42235	5.734869
H	-2.20521	-0.63083	5.631856
H	-0.68933	-1.25795	6.286984
H	-1.03311	0.478049	6.353063

$^{5}R_{2}$

Fe	-0.09647	-0.33928	0.20448
N	-0.13012	-0.50557	2.36457
C	0.843007	-0.89506	3.19851
N	0.396669	-0.83048	4.485679
C	-0.92504	-0.3783	4.493532
C	-1.23659	-0.18067	3.165463
H	1.83116	-1.21388	2.917558
H	0.935096	-1.0687	5.306775
N	-2.21369	-0.31067	-0.18844
C	-2.98628	-1.39534	-0.34306
N	-4.28618	-1.01323	-0.48739
C	-3.07125	0.800471	-0.23814
C	-4.3698	0.380857	-0.42509
C	-5.65526	1.128023	-0.55129
H	-6.13543	0.950106	-1.5207
H	-5.47792	2.202327	-0.46375
H	-2.70202	1.805362	-0.13359
H	-2.64806	-2.4167	-0.34732
H	-5.07	-1.63706	-0.61854
H	-6.36887	0.844972	0.231342
N	0.201938	-2.45154	-0.21814
C	0.603618	-2.9752	-1.3853
N	0.664798	-4.33408	-1.28328
C	0.287108	-4.71599	0.006628
C	0.001	-3.53282	0.653592
C	0.25023	-6.13766	0.458988
H	1.240449	-6.60581	0.409692
H	-0.43418	-6.7415	-0.14831
H	-0.32416	-3.38477	1.668522
H	0.828104	-2.42527	-2.28214
H	0.938379	-4.96764	-2.02125
H	-0.09025	-6.19452	1.495394
O	0.073579	0.280631	-1.91229
H	0.899435	0.53258	-2.36142

H	-0.71457	0.40231	-2.40689	H	-0.69756	0.336746	-2.50407
H	-2.24356	0.276989	2.643194	H	-2.1677	0.146842	2.735878
C	-1.8196	-0.09106	5.650715	C	-1.7175	-0.19414	5.744484
H	-2.80149	0.322475	5.409795	H	-2.71837	0.1726	5.505877
H	-1.97983	-1.02665	6.199209	H	-1.83227	-1.13512	6.295182
H	-1.321	0.611912	6.328273	H	-1.25014	0.532549	6.419314
O	0.110016	1.924064	0.65413	O	0.011983	1.853154	0.651979
H	0.057951	2.682075	0.042671	H	0.01383	2.584331	0.008539
H	-0.14113	2.193145	1.557685	H	-0.23258	2.163374	1.542961
O	1.901687	-0.14002	0.216499	O	2.069272	-0.07359	0.171555
H	2.231908	0.696761	0.588989	H	2.464216	0.753567	0.500501
H	2.561604	-0.85312	0.197321	H	2.735631	-0.76893	0.030294

$^5R_1$

C	1.126225	3.462430	12.136492
N	-0.237355	3.359630	11.967171
C	1.469215	4.682095	11.618619
C	-0.678237	4.487068	11.370327
N	0.333813	5.319148	11.141324
C	3.927674	8.765419	12.135739
N	2.945672	9.206758	12.996532
C	3.267817	8.115875	11.128597
C	1.747555	8.827542	12.507414
N	1.901756	8.157707	11.367614
C	-2.851798	8.557923	12.769985
N	-3.061099	9.330798	11.648059
C	-1.763214	7.779571	12.481477
C	-2.123271	9.012899	10.732062
N	-1.311652	8.068535	11.202035
O	-0.927405	6.633894	8.532032
H	-0.529820	5.944005	7.973664
H	-1.867495	6.405333	8.621634
H	3.674534	7.628566	10.256074
H	3.094253	9.726742	13.852706
H	0.808401	9.047466	12.992671
H	2.442460	5.148231	11.575839
H	-0.814172	2.574516	12.243478
H	-1.716131	4.662805	11.128718
H	-1.283526	7.032454	13.096190
H	-3.792888	10.020786	11.528903
H	-2.060171	9.481590	9.761349
Fe	0.374841	7.239075	10.195056

O	1.821473	6.341734	8.745842
H	2.436461	5.602553	8.883425
H	2.254501	6.952650	8.126449
O	0.688296	8.903751	8.731988
H	0.917242	9.816795	8.972460
H	0.049998	8.962292	8.001567
H	4.975580	8.949547	12.315597
H	-3.480605	8.632025	13.643878
H	1.708704	2.680184	12.598179

(1) Simmons, C. R.; Hao, Q.; Stipanuk, M. H. *Acta Crystallogr., Sect. F: Struct. Biol. Cryst. Commun.* **2005**, *61*, 1013.

(2) (a) Schmidt, T. G. M.; Skerra, A. *Nat. Protoc.* **2007**, *2*, 1528; (b) Skerra, A.; Schmidt, T. G. *Methods Enzymol.* **2000**, *326*, 271.

(3) Tchesnokov, E. P.; Wilbanks, S. M.; Jameson, G. N. L. *Biochemistry* **2012**, *51*, 257.

(4) (a) Dominy, J. E., Jr.; Hwang, J.; Guo, S.; Hirschberger, L. L.; Zhang, S.; Stipanuk, M. H. *J. Biol. Chem.* **2008**, *283*, 12188; (b) Kleffmann, T.; Jongkees, S. A.; Fairweather, G.; Wilbanks, S. M.; Jameson, G. N. L. *J. Biol. Inorg. Chem.* **2009**, *14*, 913.

(5) Bollinger, J. M.; Tong, W. H.; Ravi, N.; Huynh, B. H.; Edmonson, D. E.; Stubbe, J. *J. Am. Chem. Soc.* **1994**, *116*, 8015.

(6) Price, J. C.; Barr, E. W.; Tirupati, B.; Bollinger, J. M.; Krebs, C. *Biochemistry* **2003**, *42*, 7497.

(7) Siakkou, E.; Wilbanks, S. M.; Jameson, G. N. *Anal. Biochem.* **2010**, *405*, 127.

(8) Copeland, R. A. *Enzymes: A Practical Introduction to Structure, Mechanism, and Data Analysis* 2nd ed.; Wiley-VCH: New York, 2000.

(9) Li, W.; Blaesi, E. J.; Pecore, M. D.; Crowell, J. K.; Pierce, B. S. *Biochemistry* **2013**, *52*, 9104.

(10) (a) Kleffmann, T.; Jongkees, S. K.; Fairweather, G.; Wilbanks, S.; Jameson, G. L. *J. Biol. Inorg. Chem.* **2009**, *14*, 913; (b) Siakkou, E.; Rutledge, M. T.; Wilbanks, S. M.; Jameson, G. N. L. *Biochim. Biophys. Acta* **2011**, *1814*, 2003.

(11) (a) Dominy, J. E.; Hwang, J.; Guo, S.; Hirschberger, L. L.; Zhang, S.; Stipanuk, M. H. *J. Biol. Chem.* **2008**, *283*, 12188; (b) Njeri, C. W.; Ellis, H. R. *Arch. Biochem. Biophys.* **2014**, *558*, 61.

(12) Davies, C. G.; Fellner, M.; Tchesnokov, E. P.; Wilbanks, S. M.; Jameson, G. N. L. *Biochemistry* **2014**, *53*, 7961.

(13) Aluri, S.; de Visser, S. P. *J. Am. Chem. Soc.* **2007**, *129*, 14846.

(14) Jaguar; version 7.9 ed.; Schrodinger, LLC: New York, 2011.

(15) Frisch, M. J.; Gaussian 09; C.01; Gaussian Inc.: Wallingford, CT, 2010.

(16) (a) Becke, A. D. *J. Chem. Phys* **1993**, *98*, 5648; (b) Lee, C.; Yang, W.; Parr, R. G. *Phys. Rev. B Condens Matter* **1988**, *37*, 785.

(17) Hay, P. J.; Wadt, W. R. *J. Chem. Phys.* **1985**, *82*, 270.

(18) (a) Adamo, C.; Barone, V. *J. Chem. Phys.* **1999**, *110*, 6158; (b) Becke, A. D. *Phys. Rev. A* **1988**, *38*, 3098; (c) Perdew, J. P. *Phys. Rev. B Condens Matter* **1986**, *33*, 8822.

(19) Neese, F.; ORCA; 2.9 ed. Bonn, Germany, 2009.

(20) Faponle, A. S.; Quesne, M. G.; Sastri, C. V.; Banse, F.; de Visser, S. P. *Chem. Eur. J.* **2015**, *21*, 1221.

(21) Sinnecker, S.; Svensen, N.; Barr, E. W.; Ye, S.; Bollinger, J. M.; Neese, F.; Krebs, C. *J. Am. Chem. Soc.* **2007**, *129*, 6168.

(22) Driggers, C. M.; Cooley, R. B.; Sankaran, B.; Hirschberger, L. L.; Stipanuk, M. H.; Karplus, P. A. *J Mol Biol* **2013**, *425*, 3121.
